# Bayesian Networks in Environmental Risk Assessment: A Review

**DOI:** 10.1002/ieam.4332

**Published:** 2020-10-06

**Authors:** Laura Kaikkonen, Tuuli Parviainen, Mika Rahikainen, Laura Uusitalo, Annukka Lehikoinen

**Affiliations:** ^1^ Ecosystems and Environment Research Programme University of Helsinki Helsinki Finland; ^2^ Helsinki Institute of Sustainability Science University of Helsinki Helsinki Finland; ^3^ Bioeconomy Statistics Natural Resource Institute Finland Helsinki Finland; ^4^ Programme for Environmental Information Finnish Environment Institute Helsinki Finland; ^5^ Kotka Maritime Research Centre Kotka Finland

**Keywords:** Causal inference, Decision support, Integrated modeling, Risk analysis

## Abstract

Human activities both depend upon and have consequences on the environment. Environmental risk assessment (ERA) is a process of estimating the probability and consequences of the adverse effects of human activities and other stressors on the environment. Bayesian networks (BNs) can synthesize different types of knowledge and explicitly account for the probabilities of different scenarios, therefore offering a useful tool for ERA. Their use in formal ERA practice has not been evaluated, however, despite their increasing popularity in environmental modeling. This paper reviews the use of BNs in ERA based on peer‐reviewed publications. Following a systematic mapping protocol, we identified studies in which BNs have been used in an environmental risk context and evaluated the scope, technical aspects, and use of the models and their results. The review shows that BNs have been applied in ERA, particularly in recent years, and that there is room to develop both the model implementation and participatory modeling practices. Based on this review and the authors’ experience, we outline general guidelines and development ideas for using BNs in ERA. *Integr Environ Assess Manag* 2021;17:62–78. © 2020 The Authors. *Integrated Environmental Assessment and Management* published by Wiley Periodicals LLC on behalf of Society of Environmental Toxicology & Chemistry (SETAC)

## KEY POINTS

- This paper reviews the use of Bayesian Networks in Environmental Risk Assessments (ERA) based on peer‐reviewed publications.
- The review shows that BNs have been applied in ERA particularly in recent years and that there is room to develop both the model implementation and participatory modeling practices.
- Based on this review and the authors' experience, we outline general guidelines and development ideas for using BNs in ERA.

## INTRODUCTION

Environmental risks emerge when human activities have adverse impacts on the environment. Societies and their interaction with ecosystems are called social‐ecological systems (SES), which are often highly complex (Ostrom 2009). This complexity creates uncertainty about the risks. To assess and control these risks, we need to understand the factors contributing to the likelihood and magnitude of the adverse impacts.

**Figure 1:**
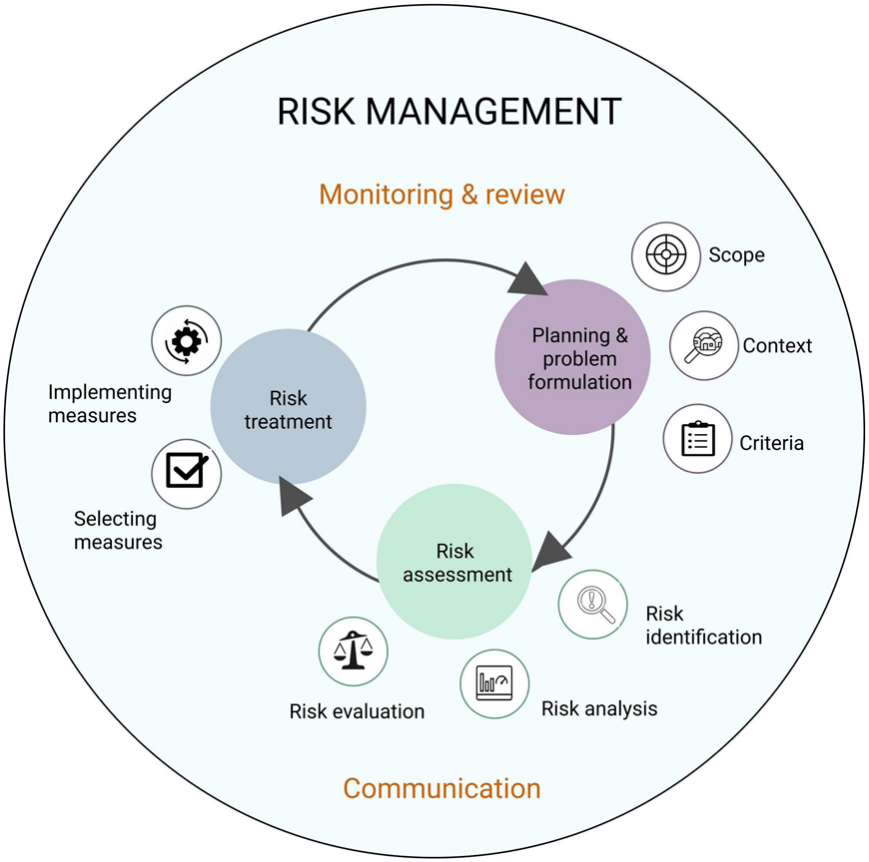
Risk assessment as a part of risk management (adapted from ISO 2018 and EPA 2018). Planning and problem formulation include setting the scope, context, and criteria for risk assessment. Risk assessment includes the stages of risk identification, risk analysis (the quantification of risks), and risk evaluation (consideration and comparison of risk reduction measures). Risk treatment includes selecting and implementing treatment measures. The risk management process is an iterative process based on continuous monitoring and review as well as communication.

Environmental risk assessment (ERA) is a process of estimating the probability and consequences of the potential adverse effects of human activities on the environment (USEPA 1998; Jardine et al. 2003; Burgman 2005). By evaluating the nature and extent of the uncertainties, ERA aims to provide a plausible and justified picture of the possible outcomes of human activities and future management actions (Ascough et al. 2008; Fenton and Neil 2012). As management interventions are directed toward unknown future conditions, it is essential to identify the probable future outcomes with tools that are robust under uncertainty (Schindler and Hilborn 2015). An ideal ERA model should thus allow exploring, explaining, and forecasting the responses of an environmental system to changes in natural and human‐induced stressors in the presence of incomplete knowledge (McIntosh et al. 2011; Whelan et al. 2014). Although the terminology, scope, and elements vary among the existing ERA frameworks (see Cains and Henshel [this issue]), the fundamental purpose of the ERA process is—through risk identification, analysis, and evaluation—to find optimal management actions under uncertainty (Figure 1).

Bayesian networks (BN) offer a useful tool for ERA because they can integrate different types of knowledge, logic, and rules in systemic entities. In a BN model, dependencies between the variables are represented as conditional probability distributions, explicitly addressing uncertainty in different parts of the analyzed system. BNs can be extended into influence diagrams (ID) that include the different decision options and valuations of the various outcomes. These models can be used in decision analysis to find the formally optimal management strategies under different scenarios.

Since their first applications in environmental sciences at the end of the 20^th^ century (Varis and Kettunen 1988; Varis and Kuikka 1997; Reckhow 1999), BNs have been gaining more popularity. In 2011, Aguilera and coauthors reviewed how BNs had been used in environmental modeling; to our knowledge, this is the only general literature mapping study on BNs in the field of environmental sciences so far. Environmental BN studies have since been reviewed in the context of climate change (Sperotto et al. 2017), water resource management (Phan et al. 2016), ecological risk assessment for freshwater and estuarine ecosystems (McDonald et al. 2015), and ecosystem service modeling (Landuyt et al. 2013). Although these reviews cover many key aspects of BN modeling, the use of BNs in ERA has not been previously evaluated. Therefore, we conducted a systematic literature mapping study (James et al. 2016) in Scopus and Web of Science to evaluate how BNs have been used in ERA, based on peer‐reviewed literature. The identified articles (497) were screened for relevance at the title, abstract, and full text level using predefined inclusion criteria. Through an iterative framing process, 72 studies presenting BN models were included for further content analysis to evaluate their contribution to ERA.

The present paper is structured as follows: After outlining the properties of BNs, we provide details of the literature search and screening procedures, followed by a description of the data extraction protocol. In the *Results* section, statistics of the search and screening procedures followed by a critical appraisal of the process are presented. In the *Discussion* section, we elaborate the primary research question on the use of BNs in an ERA context, further reflecting the results to our own experiences on applying the methodology, and acknowledging also the latest methodological advancements in the field of BN modeling.

## BAYESIAN NETWORKS

Bayesian networks (BN, also called belief networks or Bayesian belief networks), are a type of a probabilistic model consisting of 1) a directed acyclic graph defining the conditional dependencies (and, by implication, independencies) between the variables (often called nodes), and 2) the strength and shape of these dependencies as quantified by conditional probabilities (Pearl 1986) (the basic principles of BNs are illustrated in Figure S1). A directed acyclic graph indicates that the links between the variables are directed, that is, “arrows” from one variable to another, and acyclicity means these arrows are not allowed to form a loop. Introduction to BNs in the risk assessment context is given by Fenton and Neil (2012). Key textbooks on the method include those by Jensen and Nielsen (2007), Kjaerulff and Madsen (2008), and Korb and Nicholson (2010).

Both the structure and the parameters of a BN can be defined either by using algorithms to derive them directly from data or through expert judgment (potentially using previous research, data, literature, etc.). Learning the structure from data is computationally challenging; the algorithms need ample data and generally must rely on heuristics or constraints to assist the structure search (Barber 2012). Once the structure is defined, expectation maximization algorithms (Dempster et al. 1977; Lauritzen 1995) may be used to iteratively learn the parameter values from data, even if some data are missing.

When the BN structure is defined by experts, it usually aims to mimic the known causal relationships in the modeled system. This causal approach enables the evaluation of cascading effects through the system and of potential factors that may increase or reduce the risks (Fenton and Neil 2012; see the oil spill example in Supplemental Data Figure S2). A qualitative causal representation alone can help us understand how risks emerge and can be controlled (Chen and Pollino 2012; Carriger et al. 2018), making BNs a useful tool for the risk framing and identification phase of the ERA process (Figure 1). These models can also be changed into IDs by augmenting them with variables enumerating the decision options and representing the values related to the different outcomes (e.g., the economic losses related to an adverse effect, or the economic or cultural value of a healthy environment; Kjaerulff and Madsen 2008).

As probabilistic models, the result of the BNs is a distribution over the possible values of each variable, which allows the assessment of not only the expected (average) or most likely outcome but also the uncertainty associated with the prediction (Fenton and Neil 2012). For example, a model could assess the probability that a fish stock size will collapse below a critical limit under different scenarios (Uusitalo et al. 2012), or that an ecosystem reaches an acceptable status in terms of a set of ecological indicators (Moe et al. 2016).

BNs can therefore be regarded as a scenario synthesis tool, in which all possible combinations of events are taken into account by weighting them according to how likely they are to occur (Pihlajamäki et al. 2020). Value of information analysis can be used to compute the expected economic value of knowing the state of a variable before deciding about the risk controlling strategy, if the model includes economic values for the interest variables (Mäntyniemi et al. 2009). Entropy‐based sensitivity analysis, in turn, helps recognize variables that have the greatest information value for predicting the status of the assessment endpoints (Lehikoinen et al. 2019). This type of information can also be used to support rational allocation of the restricted resources for monitoring and research (Morgan 2005).

The modular nature of the BNs enables the combining of multiple networks, supporting iterative model development. Networks with at least 1 identical node can be interlinked to form a more holistic system, supporting integration of modeling work as done, for example, in separate projects. The integration starts a 2‐way information flow between the subsystems, which may provide interesting insights on how they are interrelated. This relationship is based on the BN ability to support bidirectional reasoning, both predictive from causes to effects and diagnostic from observations to their potential causes (Korb and Nicholson 2010; Carriger et al. 2016).

According to decision theory (Raiffa and Schlaifer 1961), the best management action is the one that maximizes the total expected utility while minimizing the (potential) losses (Fenton and Neil 2012). However, sometimes the scenario producing the highest expected utility may also bear the greatest uncertainty concerning its output, including the possibility of failing to meet management objectives. At the same time, a scenario with smaller expected utility may operate through well‐known mechanisms, thus resulting in smaller uncertainty and a lower probability for failure. This transparent notion of uncertainty related to the results of risk evaluation is another asset of BNs as a tool for ERA.

## METHODS

Article screening was conducted following the protocol of the open access online tool CADIMA (Kohl et al. 2018; www.cadima.info), developed to assist the working of systematic mapping and review teams. The content analysis was conducted using a questionnaire developed by the authors (Supplemental Data S4).

### Search strategy

Literature searches were conducted from Web of Science and Scopus in May–June 2019. The search strings for different combinations of “Bayesian,” “network,” “ecological,” “environmental,” and “risk” (see Supplemental Data Table S3) were used to find the studies considering the BNs in the ERA context. The searches included titles, abstracts, and keywords of the articles*.* The inclusion of gray literature such as project reports was outside the scope of this review, but this would be a valuable addition in the future to evaluate the practical applications of BNs in ERA.

### Study selection

#### Step 1: Screening of the title and abstract

After duplicate removal, records were screened based on the titles and abstracts, using the following preliminary inclusion criteria:

1. The record is a scientific article, published in a peer‐reviewed journal.
2. The record is said to present (develop, apply, or analyze) a BN model.
3. The analytical question of the study relates to ERA, wherein “environment” refers to the living environment of humans or wildlife.

Each article was evaluated independently by 2 authors. In a case of inconsistency between judgments of the 2 authors, all authors discussed it and decided together whether to exclude the paper or pass it on to the next phase.

#### Step 2: Full text screening

We used the following specifications for the full text screening step:

1. Concerning the definition of a BN, we decided to include all articles presenting a model that was defined as a BN, if it met all other inclusion criteria.
2. The article should provide at least 1 application of a BN model, explaining its principle, structure, data, or other sources of background information.
3. Regarding the concept of environmental risk, articles focusing on purely the occurrence of natural phenomena (e.g., landslides, storms, floods), not considering environmental impacts or human impact on their occurrence, were excluded. Articles focusing only on human health issues were not in the scope of the present analysis.

**Figure 2:**
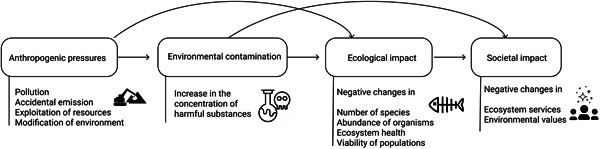
Conceptual diagram of how environmental risk analysis (ERA) is considered in the present literature mapping study. ERA is thought to operationalize when at least one of the links (arrows) connecting the human society with the ecosystems is analyzed (modeled). List of examples is not exhaustive.

Because many of the included papers mentioned the connection of the study to the ERA only in the abstract and introduction without actually contributing to the context in practice, the need for a more precise definition of the ERA concept was recognized. The outcomes of this discussion are synthesized in Figure 2, stating that the ERA studies in the focus of this literature mapping study analyze the likely extent of harmful environmental effects caused by human activities, and further on, the likely extent of impacts of these environmental changes on the society.

### Content analysis

To evaluate how the selected BN models had been applied in an ERA context, we developed a questionnaire to identify both methodological and thematic attributes of the models (Supplemental Data S4). Each paper was analyzed by 1 author. The questionnaire includes both multiple choice and open‐ended questions concerning these points:

- The purpose of the presented analysis and model, and their contribution to ERA
- The model building process
- Presented analytics
- The intended end use of the model and its results
- The pros and cons of the methodology mentioned in the article and development ideas.

## RESULTS

### Article screening

**Figure 3:**
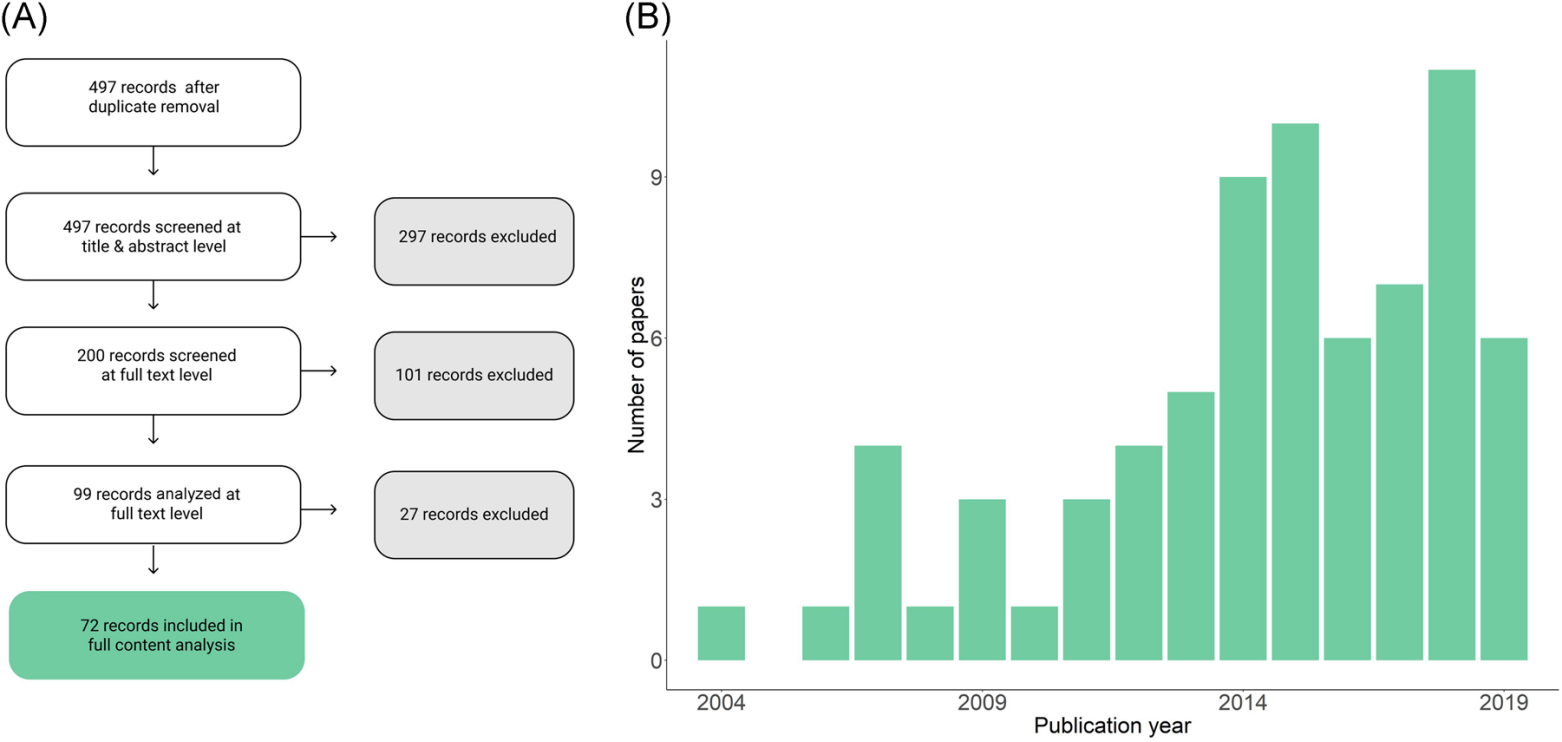
Literature screening process for the analysis (**A**) and papers included in the content analysis by publication year (**B**).

The search procedure resulted in a total of 497 records (Figure 3). Full content analysis was conducted for 72 studies. All analyzed papers are relatively recent, with more publications on the subject in recent years (Figure 3). The oldest publications included in the analyses based on the inclusion criteria were from 2004.

Despite our preceding conceptual framing exercise (Figure 2), the most difficult issue in the article selection process was the wording “ERA context” in our research question. A key topic was whether to include a paper that we thought would be useful for ERA purposes, and where some of the links of Figure 2 are covered, but where the authors of the analyzed paper do not clearly present their BN as a model to be used for ERA (see Tables S5 and S6 in Supplemental Data for a list of studies and reasons for exclusion). However, in some cases this was a fine line to draw and may have resulted in variability among the analysts. The studies excluded before content analysis consisted of 15 articles concerning human health issues and 32 with other types of environments (e.g., built environments and industrial risks) (Table S6).

### Scope and purpose of the analyzed models

The BN models selected for analysis came from multiple domains, including hydrology, fisheries science, ecology, agricultural science, environmental toxicology, and environmental chemistry (Table 1). Given the complexity of environmental risks, many studies were interdisciplinary, making any classification reductionist. Water quality was included as an assessment endpoint in 8 studies. Biological invasions were another risk factor covered in several studies, estimating, for example, the probability of establishment and ecological effects of nonindigenous invasive species (Herring et al. 2015; Lohr et al. 2017). The majority of studies covered risks in the freshwater (24 papers) and marine (23 papers) environments, with terrestrial and urban studies less well represented (Table 1).

**Table 1:** Analyzed articles by principal field of science and domain

Field	Domain	References
Ecology	Terrestrial and urban	Ayre and Landis (2012), Martin et al. (2015), Benjamin‐Fink and Reilly (2017), Lohr et al. (2017), Li et al. (2018), Ng et al. (2018), Weyer et al. (2019)
	Freshwater	Pollino et al. (2007), Roberts et al. (2013), Ayre et al. (2014), Boets et al. (2015), Perez‐Minana (2016), Shan et al. (2019)
	Marine, coastal, and estuarine	Hamilton et al. (2007), Gibbs (2007), Ban et al. (2015), Herring et al. (2015), Maxwell et al. (2015), Helle et al. (2016), Wooldridge et al. (2017), Wu et al. (2018), McDonald et al. (2016), Graham et al. (2019)
Ecotoxicology and environmental chemistry	Terrestrial and urban	Voie et al. (2010), Bayliss et al. (2012), Carriger and Newman (2012), Tighe et al. (2013), Albuquerque et al. (2017)
	Freshwater	Money et al. (2014), Money et al. (2012), Harris et al. (2017), Landis et al. (2017), Johns et al. (2017)
	Marine, coastal, and estuarine	Helle et al. (2011), Carriger and Barron (2011), Arzaghi et al. (2018), Zhang et al. (2018), Fahd et al. (2019), Liu and Callies (2019), Lu et al. (2019)
Engineering, logistics, and technology	Terrestrial and urban	Shandilya et al. (2018), Malekmohammadi and Moghadam (2018)
	Freshwater	—
	Marine, coastal, and estuarine	Klemola et al. (2009), Leiger et al. (2009), Montewka et al. (2013), Jolma et al. (2014), Lehikoinen et al. (2015), Ayele et al. (2016)
Environmental management and economics	Terrestrial and urban	Newton et al. (2007), Grêt‐Regamey et al. (2013)
	Freshwater	Barton et al. (2008), McVittie et al. (2015)
	Marine, coastal, and estuarine	Stelzenmüller et al. (2011), Fletcher et al. (2014), Rahikainen et al. (2014), Helle et al. (2015)
Fisheries and aquaculture	Freshwater	Borsuk et al. (2006), Hines and Landis (2014), Wyman‐Grothem et al. (2018)
Hydrology and earth sciences	Terrestrial and urban	Nash et al. (2013), Subagadis et al. (2014), Bashari et al. (2016), Garcia‐Prats et al. (2018), Weil et al. (2018)
	Freshwater	Mesbah et al. (2009), Pang and Sun (2014), Shenton et al. (2014), Ahmadi et al. (2015), Van Looy et al. (2015), Maldonado et al. (2016), O'Brien et al. (2018)
	Marine, coastal, and estuarine	Borsuk et al. (2004)

Despite the broad spectrum of environmental risks and the case studies in the analyzed models, most of the studies can be divided into approaches wherein the aim is to assess 1) the risk of a specific stressor to the environment in general, 2) the risk from a variety of stressors to a specific area, habitat, or species, or 3) both (Table 2). Many studies included multiple endpoints and risk sources, and the division presented here is not exhaustive.

**Table 2:** Environmental risk focus of the analyzed articles with examples

Risk of	References
Toxic substances: contamination and exposure	Albuquerque et al. (2017), Ayele et al. (2016), Bayliss et al. (2012), Fahd et al. (2019), Harris et al. (2017), Landis et al. (2017), Liu and Callies (2019), Money et al. (2014, 2012), Shandilya et al. (2018), Tighe et al. (2013), Voie et al. (2010)
Project, activity, event	Ahmadi et al. (2015), Malekmohammadi and Moghadam (2018), Weyer et al. (2019), Zhang et al. (2018)
Biological invasion or toxicity	Boets et al. (2015), Hamilton et al. (2007), Herring et al. (2015), Lohr et al. (2017), Martin et al. (2015), Ng et al. (2018), Shan et al. (2019), Wyman‐Grothem et al. (2018)
Modification of environment	Bashari et al. (2016), Garcia‐Prats et al. (2018), Weil et al. (2018)
**Risk to**	
Species or communities	Pollino et al. (2007), Ayre et al. (2014), Ban et al. (2015), Benjamin‐Fink and Reilly (2017), Hines and Landis (2014), Maxwell et al. (2015), Roberts et al. (2013), Shenton et al. (2014), Wooldridge et al. (2017), Wu et al. (2018)
State of the environment	Ayre and Landis (2012), Fletcher et al. (2014), Graham et al. (2019), Johns et al. (2017), Maldonado et al. (2016), McVittie et al. (2015), O'Brien et al. (2018), Subagadis et al. (2014), Van Looy et al. (2015)
Ecosystem services	Carriger and Barron (2011), Grêt‐Regamey et al. (2013), Pérez‐Miñana (2016)

A large number of the studies concerned risks posed by toxic substances, evaluating notably the risk of environmental contamination from the pollution source to the environment (Landis et al. 2017) or the probability of the exposure and its effects on the environment (Helle et al. 2016) (Table 2).

Only a small number of models analyzed here addressed the risk arising from specific events or activities (Table 2). These risk factors from specific sources included risks arising from aquaculture development (Gibbs 2007), accidents (Zhang et al. 2018), dam construction (Ahmadi et al. 2015; Malekmohammadi and Moghadam 2018), and mine site rehabilitation (Weyer et al. 2019). Some of these studies also assessed risk to specific environmental components, and many studies addressing toxicity risks also account for accidents when evaluating the probability of the release of contaminants.

**Figure 4:**
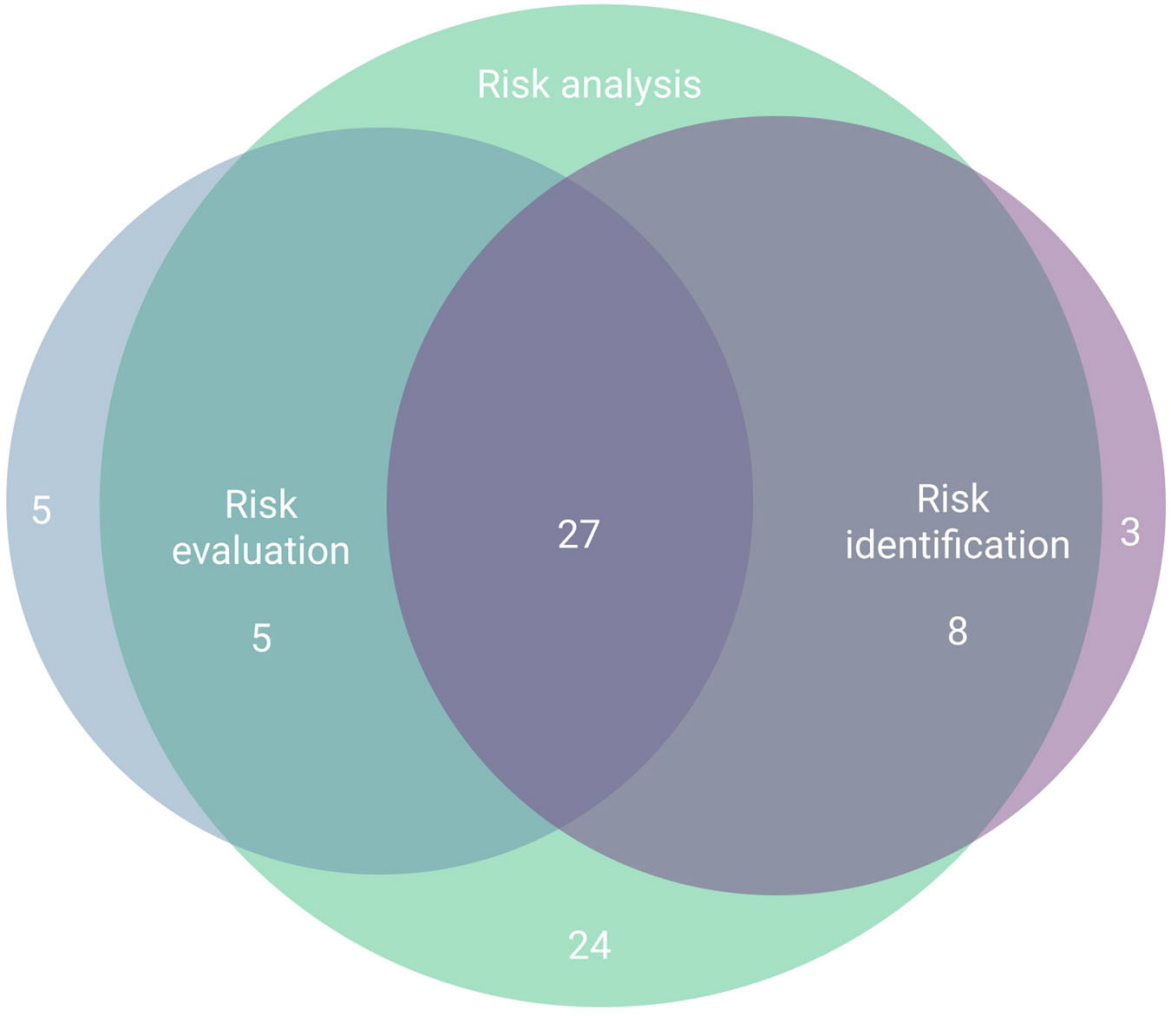
Contribution of the analyzed models to different phases of ERA.

More than 80% of the analyzed papers used BNs for risk analysis (Figure 4). Risk identification was addressed in 38 studies and risk evaluation in 36 studies, often combined with risk analysis (terms explained in Figure 1). We identified 27 studies in which BN was seen to be used to address all 3 steps of the ERA process, from risk identification to evaluation (Figure 4, Table S7).

### Model implementation

**Figure 5:**
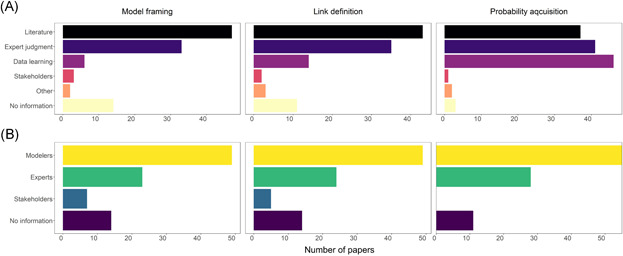
Methods (**A**) and participants (**B**) involved in the different stages of model building in the analyzed papers.

To evaluate the technical implementation of BN models in the ERA context, we analyzed both the applied methods and parties involved in different stages of modeling: model framing and variable selection, defining the structure (the arcs) between variables and their direction, and the quantification of the model through probability estimates (Figure 5). Full details of the properties of the models and other results of the content analysis are given in the Supplemental Data (Table S7).

Literature was used most often as the basis of model framing, and in the majority of the papers the model framing was done by the modeling team (Figure 5A). Similarly, model structure was most often defined through the literature by modelers, or based on expert knowledge (Figure 5). Structural learning was used in only 7 papers. Most of these studies combined structural learning algorithms with expert judgment and modified the data learnt network structure accordingly (Boets et al. 2015; Shan et al. 2019).

In most studies, probability assignment was made through a combination of expert judgment and literature, or by data‐based parameter learning (Figure 5A). External experts were involved in the probability judgments in more than 50% of the studies, and in most the modeling team had the main responsibility of the probability acquisition (Figure 5B).

### Model types

**Figure 6:**
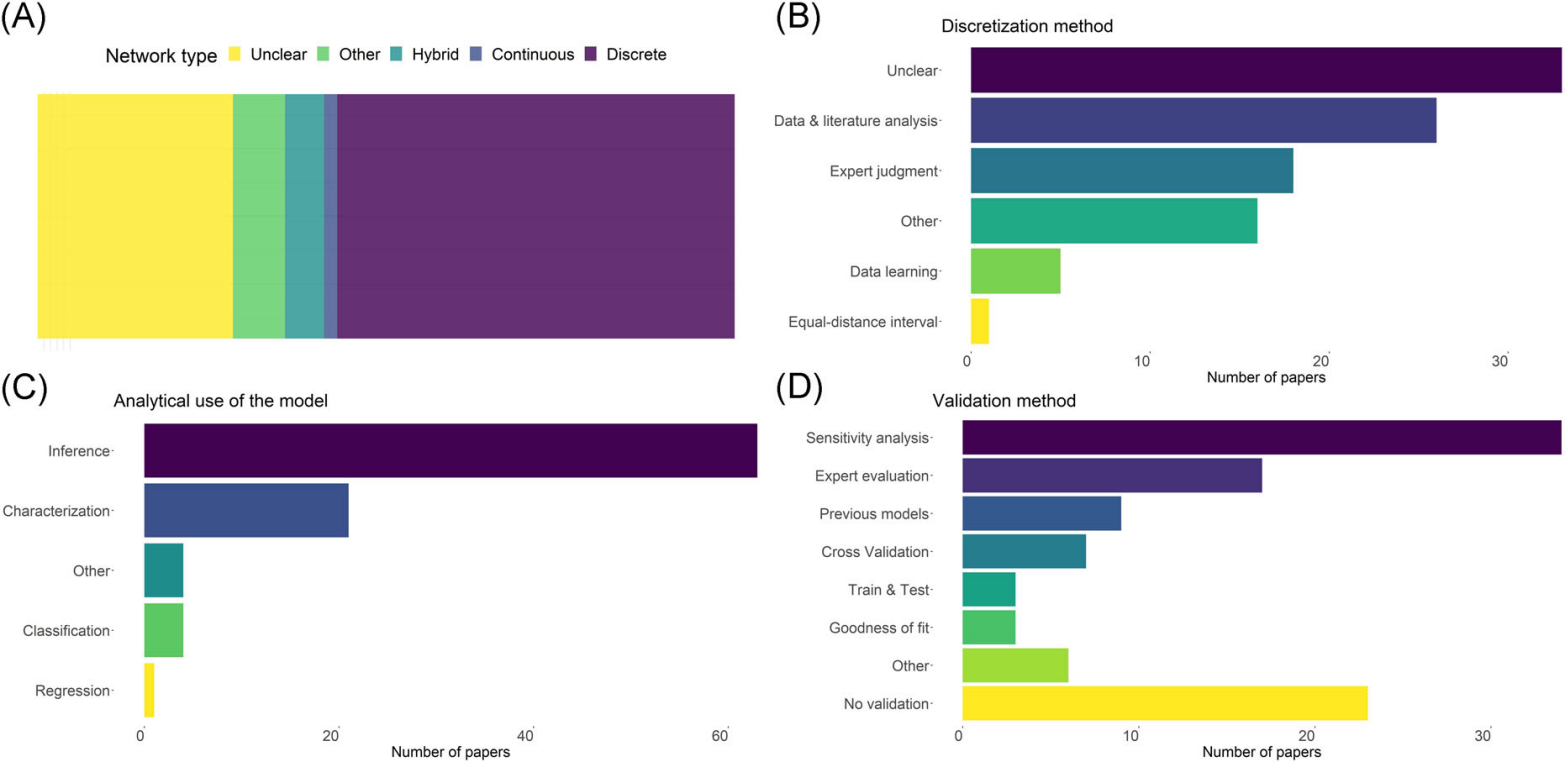
Technical properties of the analyzed BN models: (**A**) BN types, (**B**) variable discretization methods, (**C**) presented analytics, and (**D**) validation methods in the analyzed BN models.

Most of the BNs in the analyzed studies were discrete, with only 1 model identified as continuous, and 2 as hybrid networks, combining continuous and discrete nodes (Figure 6 and Table S7). Discretization was most often done through expert judgment and literature, for example, referring to policy targets and legislative boundaries for water quality (Maldonado et al. 2016). In more than 30% of the papers, no information was given on the discretization method and selection of variable states. Spatial application of the risk model was reported in 14 of the studies, either to map out the geographic extent of risk or to make use of spatial data for as predictor variables in the model (Bashari et al. 2016; Ng et al. 2018).

### Use of the BN models

The analytical focus of most models was on inference (Figure 6C), that is, predicting the values of the variables of interest by computing posterior probabilities given new evidence. Comparison to other existing models was the validation method most used (Figure 6D), followed by sensitivity analysis and cross‐validation. We note, however, that in the majority of the studies using entropy reduction and sensitivity analysis, these procedures were used as an analytical tool to evaluate the effect of external variables on the variable of interest. The aim has therefore been to map out the main factors contributing to the level and likelihood of the studied risk factor, instead of using them to validate the model.

Decision analytical elements (decision or utility nodes) were included in the BN in 18 of the 72 models. More than half these studies (10 of 18) included both, whereas the rest of the papers included only decision nodes (6) or utility nodes (2). Interestingly, many of the papers that included both decision and utility nodes focused on oil spill risks (Carriger and Barron 2011; Helle et al. 2015). Other topics included pesticide risk management (Carriger and Newman 2012), nonindigenous species management (Herring et al. 2015), and river basin management (Barton et al. 2008).

In models including decision and utility nodes, the expected losses and benefits of alternative decisions were obtained in a range of ways, yet often this distinction was not clearly indicated. Methods for defining decision and utility nodes included analysis of existing literature and expert knowledge (Carriger and Barron 2011; Arzaghi et al. 2018), previous models (Klemola et al. 2009), or learned or model‐based data (Helle et al. 2015).

End users of the model were not clearly specified in most of the studies and were typically interpreted to be either the model developers or decision makers (Supplemental Data Table S7). Decision makers were explicitly mentioned as end users of the model in one‐third of the papers examined. Stakeholders aside from decision makers were generally not mentioned. End users of model results were more specifically identified, including again decision makers, other scientists, and model developers. Here, too, other stakeholders remained unmentioned. In general, it is not stated explicitly who the decision makers are and how the models can be, or are intended to be, used. Among the analyzed papers, 2 studies reported that the developed BN model was already in use at the time of publication of the paper (Martin et al. 2015; Wyman‐Grothem et al. 2018). Several models had, however, been designed for specific case studies together with environmental managers and other stakeholders (Fletcher et al. 2014; O'Brien et al. 2018).

## DISCUSSION

### Application of BNs in environmental risk assessment studies

Human activities affect the environment in a variety of ways, resulting in diverse environmental risks. Consequently, the scientific articles identified in our literature mapping study included studies on various types of risks from multiple fields, highlighting the flexibility of BNs in environmental modeling and their broad application to risk modeling.

What is notable is that any BN—even a single pair of parent and child nodes—contains the key elements of risk, the probability of an event and its consequences, and could thus be used for risk analysis. For this reason, many published BN applications not included in this analysis could support some stages of ERA, but as they do not explicitly mention risk, they were not caught by our searches. The variety of existing ERA frameworks potentially hinders the usage of the term, especially in ecological studies, and many BN models contributing to ERA are not framed through the term risk but instead refer to environmental impacts and stressors (Allan et al. 2012; Ban et al. 2014) or to predicting the ecological status of specific habitats (Moe et al. 2016; Molina‐Navarro et al. 2020). Furthermore, as a result of the complex definitions of both the terms “risk” and “environment,” our analysis of the papers is subjective and open to interpretation.

In line with the limited use of ERA terminology, the analyzed studies rarely referred to the phases of ERA or specific frameworks. Only a few articles explicitly evaluated the ERA process (Bayliss et al. 2012; Landis et al. 2017; Arzaghi et al. 2018), whereas most papers contributed to specific parts of ERA (Figure 1). BNs analyzed here focused on risk analysis, and accordingly used the BN as a tool for inferring values of a small number of target variables, instead of a more holistic evaluation of the risks within a studied system. An important feature in many articles was the identification of key factors contributing to the magnitude and probability of the studied adverse effects, which was typically based on sensitivity analysis.

Not surprisingly, many studies considered toxic risks and environmental contamination. A focus of many ERA models on contamination risks, toxicity, or biological invasion was to assess the probability of the undesirable event, assessing the probability that a specific area will be contaminated, an invasive species will settle in an area, or a species will be affected by a specific environmental stressor. In addition, many studies included multiple endpoints, risk factors, including socioeconomic variables within the assessment (Malekmohammadi and Moghadam 2018), making use of the integrative properties of BNs. The use of cumulative risk assessments was, however, less common.

As a result, the use of BN models in ERA in addressing risks arising from projects and other specific activities was not well represented. With increasing expectations on the scientific rigor of environmental impact assessments (EIA), ERA is increasingly included in the EIA process for projects of different scale, although it is not a statutory requirement for many human activities (Suter 2016). Although a number of papers in our review addressed the impacts of point‐source contamination from oil spills (Arzaghi et al. 2018) and other contaminants (Harris et al. 2017), none of the studies used BNs for predictive risk assessments that included a comprehensive view of the risks and could inform further use in EIA. We appreciate, however, that the lack of practical applications of BNs in ERA may be a result of our search strategy. A further reason for the poor representation of cumulative risk assessments may be that assessing multiple stressors from human activities is challenging, and handling the multidimensionality of complex BNs requires particular attention during expert elicitation. Methods for reducing the number of conditional probabilities required for nodes with multiple parents have been reviewed by Zhang and Thai (2016), among others. These knowledge engineering methods merit further attention in future research, as many projects could benefit from more transparency and quantitative estimates in the ERA processes. The use of BNs would answer the call of better communicating uncertainty in ERAs within EIA (Tenney et al. 2006), yet this review indicates that BNs are not yet being applied for this purpose.

### Implications of currently used methodologies

A general framework for BN modeling consists of defining the aim of the model, building the model from the available information, and model validation, all of which can be performed in many different ways. Several papers, which are not comprehensively discussed here, already cover the technical aspects and good practice of BN modeling (Chen and Pollino 2012). Instead, we focus the implementation of BN models in respect to the ERA process and the potential implications of current methodologies for their practical use.

### Model implementation and technical aspects

Given the flexibility of BN modeling, the studied models represented a crosscut of possible technical implementation of models. The majority of the studies used BNs as expert systems, and the model structure was often defined based on existing models, literature, and expert and stakeholder knowledge. Even the studies that applied structural learning algorithms to learn the BN structure from data relied on expert views for refining the model structure, which is in line with the complexity of environmental issues, recognizing that the view of what is causing the risk and what is at risk are largely subject to individual values and preferences (Slovic et al. 2004). To be comprehensive, it is often useful for ERAs to incorporate stakeholder values and management objectives in addition to quantitative data.

The context and objectives of the risk assessment define the methods used in an ERA and to what extent they are expert‐ or stakeholder driven (Burgman 2005). Although the use of expert knowledge is widely accepted as indispensable for risk assessments (Pollino et al. 2007; Kuhnert et al. 2010), there is still room for improvement in terms of transparency regarding the source of expert knowledge. Information on who the experts are or how they were selected to be part of the process was often missing in the analyzed studies. To improve the legitimacy and trust in ERA, adhering to set guidelines for expert elicitation is recommended (O'Hagan et al. 2006; Kuhnert et al. 2010; Martin et al. 2012).

Most studies used only discrete variables in the BN. However, the method or criteria behind the discretization were not often detailed. Discretization of a continuous variable simplifies the probability distribution and therefore necessarily causes some loss of information (Uusitalo 2007), and in turn this may substantially affect the model outputs. For this reason it is suggested that discretization be used with caution or be avoided whenever possible (Nojavan et al. 2017). However, it is also suggested that the deviating patterns among differently discretized models can provide important information about the resolution of the covariance among variables, as well as about the potentially meaningful change points in the data (Lehikoinen et al. 2019). A straightforward solution for avoiding discretization is to simply accommodate only continuous variables in the modeling framework (Qian and Miltner 2015).

Despite acknowledging the challenges of discrete networks, continuous networks were very rare in the analyzed studies, as also found earlier in the review by Aguilera et al. (2011). As environmental management and risk modeling often require integrating both numerical and categorical variables by including, for example, management thresholds for the target variables, decision, or utility nodes, and management interventions using only continuous variables are not always ideal (Ropero et al. 2014) or even possible. Although highlighted as a feasible solution for combining discrete and continuous variables (Chen and Pollino 2012), the use of hybrid networks in environmental applications, including the studies reviewed here, is scarce. This lack is likely a result of the history of using specific BN software products that are designed for discrete variables or provide only a limited selection of continuous distribution types. Several popular BN softwares nowadays accommodate hybrid networks (e.g., Hugin, Analytica, AgenaRisk, *bnlearn* in R), but their analytical use is still limited compared to discrete models (see, however, Moe et al. 2020, for example), and requires more statistical expertise from both the modeler and the end user of the model or its results. It is also noteworthy that although conditional probability tables of discrete variables are able to accommodate and express nonlinearities, step functions, and other nonparametric functional responses between the variables, the continuous variables are often modeled using linear functions or other simple parametric functional forms. Therefore, not discretizing the variables might avoid information loss at that stage, but information loss may occur in the parameterization stage from the restrictions imposed by fitting these parametric distributions. The loss of information in discretized models can be minimized through selecting class boundaries that maximize the predictive capacity of the model or otherwise represent relevant changes in the system, for example, in terms of the (management) scenarios of interest and the potential target or limit values already set by the society. To conclude, discretization of the continuous variables, if applied, should be carefully considered and justified.

Compared to learning the BN structure from data, variable parameterization from data was more common in the analyzed studies (McDonald et al. 2016; Graham et al. 2019). As combining knowledge from multiple sources and being able to operate with missing data is an important asset of BNs and often a key reason for using them, many studies combined data learning with expert judgment. Expert knowledge was used to quantify a large portion of the analyzed studies, using a number of elicitation techniques. However, a surprisingly large number of studies did not specify the method of eliciting probabilities from experts or other sources. Using a structured approach for the expert elicitation ensures all parts of the modeling to be methodologically robust. Several guides have been published on the best practice of eliciting expert knowledge for BNs (Kuhnert et al. 2010; Werner et al. 2017), which may be consulted for improved transparency and documentation of the parameterization process.

As also noted by Aguilera et al. (2011), a high portion of the studies still did not validate the BN model. Among the popular validation methods, sensitivity analysis was often used not in the validation sense but as an analytical tool. A large number of studies used expert or stakeholder knowledge in the model building but did not report on validation measures to check the final logic of the model outputs. Validation thus does not seem to be the routine part of BN modeling that it should be. Echoing previous reviews on best practices (Aguilera et al. 2011; Chen and Pollino 2012), we encourage model developers to select a validation method that fits the objective of the modeling process. Validation approaches for expert‐driven BNs are presented by Kleemann et al. (2017), as well as others.

### Participatory modeling

The complex socioecological nature of environmental risks means that problem structuring is linked with the parties involved in the risk assessment process. Model framing is often not described in the analyzed studies, although defining the purpose and end use of the model before modeling is important for ensuring its usefulness (Chen and Pollino 2012). Including stakeholders throughout the process is vital, especially in the early stages of ERA, as the risk framing determines the rest of the process, such as who is involved in assessing risks and how this is done (Brugnach et al. 2011; Parviainen et al. 2019). BNs have been reported as beneficial for participatory modeling, as they can support open discussion between stakeholders as well as coproduction and codesign of the network structure, promote social learning (Barton et al. 2012; Henriksen et al. 2012), and increase transparency about the model structure (Henriksen et al. 2007).

The review, however, indicates a limited role of stakeholders in the process in terms of framing, defining the structure, and quantifying the models. Many of the papers analyzed in our literature review did not specify the rationale for model framing, variables selection, or model structure. Model endpoints, selection of variables, and their states are critical aspects in estimating the magnitude of the risk in question, but they are often neglected in the reporting of BN models.

BNs can be developed into an ID by including decision and utility nodes, but the results demonstrate the use of these decision analytical elements is limited. Further, how the nodes were determined is not always clear. As decision makers were often mentioned as the potential end users of the model results, their inclusion in also identifying the decision and utility nodes would be important in terms of making models more meaningful and useful for the end users. Although expert judgment was used in some cases, the results indicate that other stakeholders were not included in the process of determining the decision‐analytical elements.

### Pros and cons of BNs in ERA

To understand the perceived benefits and restrictions of BNs in ERA, we took a note of any pros and cons of BNs mentioned in the reviewed papers. The most common advantages related to the use of BNs in ERA in the reviewed studies include the explicit treatment of uncertainty, the ability of BNs to integrate knowledge from different sources, and the means to easily update the models as new knowledge becomes available. Although mentioned in only a few of the papers, BNs can also be applied to integrate socioeconomic data in addition to purely environmental or ecological data (Fletcher et al. 2014). Combining BNs with spatial data was seen to support the spatial assessment of risks as well as to improve the user experience (Jolma et al. 2014). Some of the papers also highlighted BNs as a useful tool in adaptive management (Ayre and Landis 2012; Shenton et al. 2014).

The modular nature of BNs enables building large entities piece by piece by adding new variables or connecting whole BN models with each other to form a larger entity (Van Looy et al. 2015). BNs are seen as advantageous in supporting continuous learning processes: the method enables building large entities piece by piece by adding new variables or connecting whole BN models with each other to form a larger entity (Van Looy et al. 2015). This method allows long‐term development of holistic assessments that can be expanded, fine tuned, and modified as new needs or information arise. For example, an assessment focusing on estimating the probability of a harmful event, such as contamination of a watershed, can be later complemented with more environmental and socioeconomic endpoints, alternative risk control measures, and their costs, to be compared. Within a series of studies on the risks of mercury in the South River (Virginia), the assessment first focused on the ecological risks of mercury in the river environment, and the BNs were later applied to compare different management options and to assess risks to human health (Harris et al. 2017; Johns et al. 2017; Landis et al. 2017). Similarly, studies assessing oil spill risks in the Baltic Sea used previous oil spill risk BN models as building blocks to develop new models to compare different management options (Montewka et al. 2013; Helle et al. 2015; Lehikoinen et al. 2015).

Graphical representation of BNs is helpful in stakeholder involvement in the ERA process. Visual presentation of problem structuring and the quantitative results can support consensus building among stakeholder parties (Henriksen et al. 2007; Laurila‐Pant et al. 2019). As the performance of even rather complex BNs is relatively rapid, large packages of “what‐if” questions can be tested and compared within a reasonable time, which also allows for efficient working in terms of risk communication (Figure 1) to stakeholders.

Many of the papers lacked discussion on the challenges of applying BNs specifically in the ERA context. However, the acyclic nature of BNs, and the lack of temporal scale, were the 2 disadvantages most commonly mentioned. Temporal dynamics can, however, be modeled in BNs if the temporal dimension is built into the model explicitly through time steps. Building each of the time steps into the model through their own set of variables increases the size of the model and makes it visually less appealing, but enables the temporal feedback loops to be clearly articulated.

Expert elicitation was also considered as a challenge, particularly in terms of ensuring the reliability of the elicited knowledge and avoiding biases. As reflected by the limited use of validation techniques in the analyzed studies, model validation methods were often cited as a challenge in model development. The aforementioned limitations and challenges of BNs for ERA have been discussed in length by several authors (Uusitalo 2007; Phan et al. 2016; Sperotto et al. 2017). Further challenges in the use of BNs in an ERA context largely depend on the successful implementation of the models in management processes, which we discuss in the following sections.

### End use: Making models useful for decision making

Despite following best modeling practices, decision makers may still be reluctant to use the model and its outputs to inform risk management. Ensuring that the models created for ERA actually deem themselves useful can, however, be improved through a number of measures that also apply to other types of decision support models (Addison et al. 2013). These measures include not only improving model development but also further engagement with policy making and the objectives of the risk assessment.

Stakeholder objectives and concerns provide the basis for value‐focused decisions that are fundamental for environmental management (Gregory and Keeney 1994). In general, participatory modeling is resource intensive, requiring an extensive amount of time and funding, and it may be unrealistic to expect wider stakeholder participation in ERA. In comparison to some other types of participatory modeling (PM) methods that require intensive participation and commitment from the researchers and the stakeholders involved, Davies et al. (2015) suggest BNs, if used in isolation, have a low potential to integrate social values and promote social learning to address wicked socioenvironmental problems. The level of stakeholder participation needed in each ERA process requires case‐specific consideration, after which the potential of the BN as a method to support it can be evaluated. In terms of integrating social values into ERA, creative technical approaches and practical solutions could be more actively shared within the BN modeling community.

Participatory processes in the model building may, however, improve the knowledge base and social acceptance of model results and facilitate better management outcomes. Active involvement of the end users improves chances that the model will meet their needs in terms of problem framing and the questions to be answered (Laniak et al. 2013). If the end users do not agree with the data, assumptions, logic, or the methods used for the modeling, they can hardly be expected to subscribe to the results. Stakeholder dialogue can be particularly useful when ensuring the model relevance in the validation step, as it helps ensure that the users of the modeling results agree that the model represents an accurate picture of reality based on the available knowledge (Benjamin‐Fink and Reilly 2017), and are thus more likely to trust the model outputs.

The transparency and flexibility of BNs make them an attractive tool for potential end users. The ease of use may also be seen as a caveat, as it brings about a high risk of making erroneous interpretations for unsupervised use. In addition to knowing the model, the user must be familiar with at least the basics of the Bayesian inference and probability calculus. Interactive workshops are a feasible option for improving the end use of the BN‐ERA applications, in which the model developers act as facilitators by conducting the asked runs, interpreting and explaining the results, as applied in a number of studies analyzed in this review (Fletcher et al. 2014; McVittie et al. 2015).

Although the actual use of BNs requires substantial knowledge from the users, models and their outputs may be made available to stakeholders and managers by combining them with other tools and user interfaces. Piffady et al. (this issue) developed a web‐based tool coupled to a BN to assess the spatial risk of pesticide contamination in French rivers. The tool, which enables the users to run the model without coding experience, was developed in cooperation with stakeholders.

Hart and Pollino (2008) argued that all risk assessment models should be as quantitative as possible. Although presenting uncertainty in probabilistic terms is a major improvement from traditionally vague risk assessments, it is important to consider on what occasions quantitative assessments are useful for analyzing risks within socioecological systems. As suggested by Carriger et al. (2018), even purely qualitative IDs can improve understanding of policy interventions and enhance transparency. A relevant question is whether risk assessment models should also be seen as a basis for reflection and discussion rather than simply tools for quantifying risks? In the case of “wicked” socioenvironmental decision‐making problems with no unambiguously best solution (Rittel and Webber 1973), a transparent systemic model such as BN could reveal among what aspects we are actually choosing.

### Future development ideas and guidelines for use in ERA

To increase the performance, usability, and practical application of BN models, in this section we summarize a number of development ideas for their future use in ERA (Table 3). Drawing on our own experience and the analyzed studies, we further outline general guidelines for using BNs in ERA.

**Table 3:** Summary of some key development ideas for improving use of Bayesian networks (BNs) in environmental risk assessment (ERA) with references to studies acknowledging them

Topic	Recognized needs discussed in the analyzed articles
Spatial applications	Building and updating universal models with regional, local specific data (Subagadis et al. 2014; Pérez‐Miñana 2016; Harris et al. 2017).
BN extensions	Explore use of hybrid BNs, dynamic BNs, and quantum Bayesian networks.
Integrated modeling	Making further use of the modular properties of BNs and combining them to several types of modeling and methods (Stelzenmüller et al. 2011; Carriger and Newman 2012; Martin et al. 2015).
Improved validation	Increasing validation of models and improving validation methods for strength of evidence, for example (Pollino et al. 2007).
Participatory modeling	Explore potential of further engagement with external experts and stakeholders in model building (Stelzenmüller et al. 2011; Subagadis et al. 2014; Li et al. 2018).
Comprehensive models	Developing holistic assessments in terms of including a broader variety of variables types and processes for model endpoints, analyzed measures.
Transparency of methodology	Detailing methods used, data sources, participants in model framing, variable selection, and probability acquisition.
Improved monitoring and evaluation	Monitor and report results of risk management measures (Hines and Landis 2014).

A central challenge in developing practical BN applications has long been the limited capacity of BNs to account for a sufficient spatial resolution and to flexibly incorporate local data and additional precision (Maxwell et al. 2015). Building an application for the finer scale spatial analysis calls for the integration of BNs with geographic information systems (GIS), so that spatial data may either be incorporated into the BN, or vice versa. Several studies, including those analyzed here, already make use of integrating spatial data to a BN to estimate the spatial extent of risk (Bashari et al. 2016; Helle et al. 2016).

In addition to spatial modeling tools, Marcot and Penman (2019) provide an extensive review of how BNs can be joined with other tools and model frameworks for a variety of environmental assessment and management purposes. These uses include BNs to explore system dynamics (e.g., agent‐based BNs, hybrid BNs, object‐orientated BNs) and BNs to aid decision making (e.g., Bayesian decision networks, dynamic decision networks, and quantum Bayesian networks).

Many of the reviewed papers included comparison of different management measures to reduce risks, but specific decision‐analytical nodes were rarely applied. The review suggests that expanding BNs to influence diagrams and the use of decision or utility nodes could be further explored.

BNs provide a valid tool for participatory environmental modeling, but as the review demonstrates, decision makers and other societal stakeholders are generally not included in the modeling process, or the stakeholders are involved only in specific parts of the modeling but not throughout the process. Further, as most of the models focus on ecological risks, research on the socioeconomic or cultural impacts of risks is lacking. The idea of objective science largely dominates in natural sciences and the potential model end users, such as managers and decision makers, may prefer “exact” advice rather than probability distributions, particularly when assessing chemical risks in ecotoxicology, where defining thresholds for the risk quotient is often the focus of risk assessments (Carriger and Barron 2020). Therefore, communicating uncertainty to decision makers and stakeholders remains a key challenge (see also Rahikainen et al. 2014).

As models alone cannot solve policy problems, ERA models should ideally encourage knowledge exchange by combination of scientific models and social values (Borsuk et al. 2004). Further attention should also be paid to whether BNs can support the integration of social values in ERA (Davies et al. 2015) and promote learning and capacity building needed for adaptive management of socioecological risks (Nyberg et al. 2006; Henriksen and Barlebo 2008). In addition to highlighting the uncertainty related to the expected outcomes of management actions, using probabilistic approaches invites managers and decision makers to be aware of and transparent with their risk attitudes.

## CONCLUSIONS

In this review, we examined to what extent BNs have been used in the ERA context. We found that although BNs have been applied in various fields, including several types of risk factors and contexts, the method is still not very commonly used by the ERA research community. The analyzed ERA BNs mostly contributed to assessing contamination risks and ecological risks, with only a minor part of the studies addressing socioeconomic risks. We suggest the approach has potential for more holistic ERA analyses from risk identification, through risk analysis, to risk evaluation, than that for which it has been used. To advance the use of BNs in supporting real‐life risk management and risk communication, we highlight the importance of transparency in all stages of modeling and considering novel and creative ways to apply BNs in participatory modeling. In conclusion, the current use of BNs in ERA context still has strong potential for improvement, calling further attention to how BNs could support adaptive management of complex environmental risks.

## Disclaimer

The authors declare no conflict of interest.

## SUPPLEMENTAL DATA

In Figures S1–S2 we provide additional information for applications of BNs. Table S3 contains details on the search strings for the systematic literature mapping. S4 contains the questionnaire used for the content analysis of the studied articles. S5 and S6 contain information of the articles excluded at the full‐text phase of literature screening. S7 includes results from the content analysis.

## Supporting information

This article contains online‐only Supplemental Data.

Supporting information.

Supporting information.

Supporting information.

Supporting information.

Supporting information.

Supporting information.

Supporting information.

## Data Availability

Data are publicly available. Information related to the article (results of the systematic mapping) is supplied in the supplementary data. Further details are available upon request by contacting corresponding author Laura Kaikkonen (laura.m.kaikkonen@helsinki.fi).
