# Supplementary material for: Bayesian Networks in Environmental Risk Assessment: A Review
Source: Integr Environ Assess Manag. 2020 Oct 6;17(1):62–78. doi: 10.1002/ieam.4332 (PMC7821106; doi:10.1002/ieam.4332)
Supplement: Supplementary file 1 — Supporting information. [file IEAM-17-62-s001.docx]

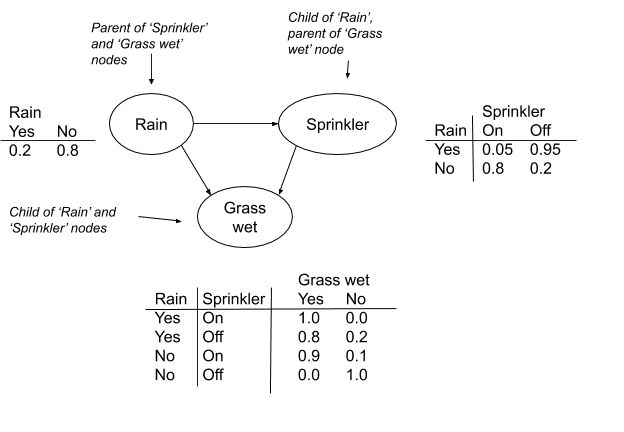


**Figure S1.** A classic example of a Bayesian network, to the best of our knowledge first presented by Pearl (1987, 1988). Beside the nodes, the probability tables of the nodes are shown. The *Sprinkler* and *Grass wet* nodes have conditional probability tables, i.e. their probability distribution depends on the state of their parent(s). In the tables of this figure, each row forms a distribution, the values representing the probabilities of the variable’s alternative states, summing up to one (i.e. 100%).The states are exhaustive, i.e. they cover all the possible outcomes, and are mutually exclusive.

**References**

Pearl J. 1987. Embracing Causality in Formal Reasoning. In: AAAI. p. 369–373.

Pearl J. 1988. Embracing causality in default reasoning. Artif Intell. 35(2):259–271. doi:10.1016/0004-3702(88)90015-X.
