# Supplementary material for: Bayesian Networks in Environmental Risk Assessment: A Review
Source: Integr Environ Assess Manag. 2020 Oct 6;17(1):62–78. doi: 10.1002/ieam.4332 (PMC7821106; doi:10.1002/ieam.4332)
Supplement: Supplementary file 2 — Supporting information. [file IEAM-17-62-s002.docx]

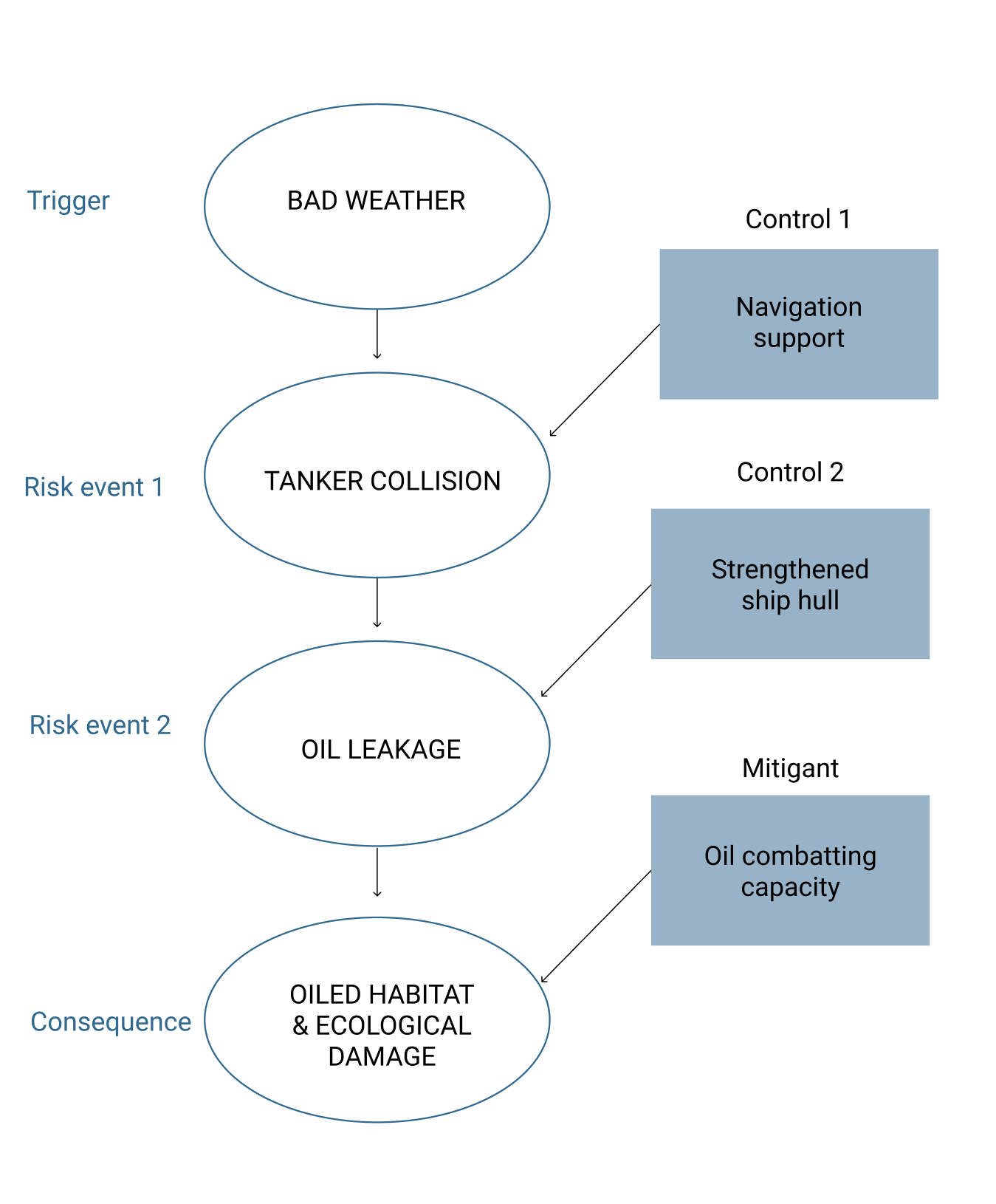


**Figure S2**. A causal chain to represent the risk of coastal contamination due to an oil spill, caused by a tanker collision, triggered by bad weather conditions (the presentation style and terminology by Fenton and Neil, 2012). As the causal dependencies (arrows) in a BN are represented in probabilistic terms (see Fig. S1), the uncertainty accumulates throughout the chain. It is also possible to add controlling and mitigating actions that affect different parts of the chain, and test and compare their effectiveness under the prevailing knowledge / uncertainty. In reality, the weather conditions strongly affect the success of oil combatting, thus an additional link from weather to ecological damage could be added.
