# Supplementary material for: Bayesian Networks in Environmental Risk Assessment: A Review
Source: Integr Environ Assess Manag. 2020 Oct 6;17(1):62–78. doi: 10.1002/ieam.4332 (PMC7821106; doi:10.1002/ieam.4332)
Supplement: Supplementary file 3 — Supporting information. [file IEAM-17-62-s003.docx]

**Table S3.** Literature searches conducted for the mapping analysis. WoS stands for the Web of Science.

| Search string | Database | Date |
| --- | --- | --- |
| "Bayesian networks" AND "ecological risk" | WoS | 2019-05-23 |
| "Bayesian networks" AND "ecological risk" | Scopus | 2019-05-23 |
| "Bayesian networks" AND "environmental risk" | WoS | 2019-05-23 |
| "Bayesian networks" AND "environmental risk" | Scopus | 2019-05-23 |
| "Bayesian network" AND "ecological" AND "risk" | Scopus | 2019-05-29 |
| "Bayesian network" AND "environmental" AND "risk" | Scopus | 2019-05-29 |
| "Bayesian" AND "network" AND "environmental risk" | WoS | 2019-06-04 |
| "Bayesian" AND "network" AND "ecological risk" | WoS | 2019-06-04 |
| "Bayesian" AND "network" AND "environmental risk" | Scopus | 2019-06-05 |
| "Bayesian" AND "network" AND "ecological risk" | Scopus | 2019-06-05 |
