# Supplementary material for: Bayesian Networks in Environmental Risk Assessment: A Review
Source: Integr Environ Assess Manag. 2020 Oct 6;17(1):62–78. doi: 10.1002/ieam.4332 (PMC7821106; doi:10.1002/ieam.4332)
Supplement: Supplementary file 4 — Supporting information. [file IEAM-17-62-s004.docx]

**S4: Questionnaire used for the analysis of the selected articles**

**Aims and scope**

1.1. Purpose and context of the model(ling)

1.1.1. Describe the research question of the paper.

1.1.2. To what question the model is intended to answer (the research question as such or some part of it)?

1.1.3. To which component(s) of risk assessment process the model contributes?

Risk identification (RI)* / Risk analysis (RA)** / Risk evaluation (decision analysis) (RE)*** / Not clear (NC)

*RISK IDENTIFICATION (ISO 31000): “The purpose of risk identification is to find, recognize and describe risks that might help or prevent an organization achieving its objectives. ... The following factors, and the relationship between these factors, should be considered: tangible and intangible sources of risk; causes and events; threats and opportunities; vulnerabilities and capabilities; changes in the external and internal context; indicators of emerging risks; the nature and value of assets and resources; consequences and their impact on objectives; limitations of knowledge and reliability of information; time-related factors; biases, assumptions and beliefs of those involved.”

**RISK ANALYSIS (ISO 31000): “The purpose of risk analysis is to comprehend the nature of risk and its characteristics including, where appropriate, the level of risk. Risk analysis involves a detailed consideration of uncertainties, risk sources, consequences, likelihood, events, scenarios, controls and their effectiveness. … Risk analysis should consider factors such as: the likelihood of events and consequences; the nature and magnitude of consequences; complexity and connectivity; time-related factors and volatility; the effectiveness of existing controls; sensitivity and confidence levels.”

***RISK EVALUATION (ISO 31000): “The purpose of risk evaluation is to support decisions. Risk evaluation involves comparing the results of the risk analysis with the established risk criteria to determine where additional action is required.”

1.2. Framing of the environmental risk in question

1.2.1. Describe the environmental risk in focus of the article (E.g. risk to what/whom, caused by what?)

**Modeling process**

2.1. Model framing

2.1.1. Who participated in the model framing (selection of the variables)?

Modeller or modelling team one-disciplinary (MO) / Modelling team multi-disciplinary (MM) / External expert or expert team one-disciplinary (EO) / External expert team multi-disciplinary (EM) / Non-expert stakeholders (SH) / No information (NI)

2.1.2. How were the variables selected?

Learned or modelled based on data (DL) / Literature-based (L) / Expert judgement (EJ) / Non-expert judgement (NJ) / Other (O) / No information (NI)

2.1.3. Describe the model framing process shortly:

2.2 Model structure

2.2.1. Who participated in defining the model structure?

Modeller or modelling team one-disciplinary (MO) / Modelling team multi-disciplinary (MM) / External expert or expert team one-disciplinary (EO) / External expert team multi-disciplinary (EM) / Non-expert stakeholders (SH) / No information (NI)

2.2.2. How were the links defined?

Learned or modelled based on data (DL) / Literature-based (L) / Expert judgement (EJ) / Non-expert judgement (NJ) / Other (O) / No information (NI)

2.2.3. How was the discretization done (if a discrete BN)?

Learned from data by an algorithm (DL) / Based on data-analysis (incl. literature) (DA) / Elicited based on expert knowledge (EE) / Elicited from non-expert stakeholders (ES) / Equal Distance Interval (EDI) / Equal Frequency Intervals (EFI) / Other (O) / No information (NI)

2.2.4. Describe the model structuring process shortly:

2.3. Probabilities

2.3.1. Is the BN Discrete (D) / Continuous (C) / Hybrid (H) / Other (O)?

2.3.2. Who participated in producing the probabilities or (if a continuous BN) the parameters of the distributions?

Modeller or modelling team one-disciplinary (MO) / Modelling team multi-disciplinary (MM) / External expert or expert team one-disciplinary (EO) / External expert team multi-disciplinary (EM) / Non-expert stakeholders (SH) / Not a quantified model (NQ) / No information (NI)

2.3.3. How were the probabilities / parameters obtained?

Learned or modelled based on data (DL) / Literature-based (L) / Expert judgement (EJ) / Non-expert judgement (NJ) / Other (O) / No information (NI)

2.3.4. Describe the probability / parameter acquisition process shortly:

2.4. Decision analytical elements

2.4.1. What decision-analytical elements the model includes?

Utility nodes (U) / Decision nodes (D) / Neither (NA)

2.4.2. Who participated in defining what utilities / losses will be considered in the model?

Modeller or modelling team one-disciplinary (MO) / Modelling team multi-disciplinary (MM) / External expert or expert team one-disciplinary (EO) / External expert team multi-disciplinary (EM) / Non-expert stakeholders (SH) / No information (NI)

2.4.3. How were the utilities / losses obtained?

Learned or modelled based on data (DL) / Literature-based (L) / Expert judgement (EJ) / Non-expert judgement (NJ) / Other (O) / No information (NI)

2.4.4. Describe the process of defining and obtaining the utilities / losses shortly:

2.4.5. Who participated in selecting the alternative decisions considered in the model?

Modeller or modelling team one-disciplinary (MO) / Modelling team multi-disciplinary (MM) / External expert or expert team one-disciplinary (EO) / External expert team multi-disciplinary (EM) / Non-expert stakeholders (SH) / No information (NI)

2.4.6. Describe the process of selecting the decision options shortly:

**3. Results**

3.1. Analysis

3.1.1. How the model is used in the study, to answer the research questions? (Options from Aguilera et al. 2011)

Inference (I)* / Characterisation (CH)** / Classification (CL)*** / Regression (R)**** / Other (O) / Not applicable (NA)

*Inference = “computed posterior probabilities, given some evidence. We call evidence or finding the knowledge about the value of one or more variables of the model. The computation of the posterior probability is called inference, evidence propagation or belief updating.” (Aguilera et al., 2011)

**Characterise = “The first result we obtain when modelling a problem through a BN is a characterisation of the actual problem, in terms of the relationships between the different variables and their strength.” (Aguilera et al., 2011) (Addition by the authors: “problem structuring”)

***Classification = “In a classification problem we are interested in predicting the value of one of the variables, usually denoted as class variable, given the values of some of the remaining variables, called feature variables. This is done via the inference process mentioned before. The main difference with respect to the above results is that our interest lies on one of the variables, and so we can concentrate and try to model as precisely as possible the relationships regarding the class variable, and not pay so much attention to the others. ... Although any BN can be used for classification, in order to stress the importance of the class variable, some fixed structure BN models are used, like the Naïve Bayes (Duda et al., 2001), Tree Augmented Network (Friedman et al., 1997) and kDB (Sahami, 1996) models.” (Aguilera et al., 2011)

****Regression = “...the goal is to predict the values of a continuous response variable given the values of some explanatory variables.” (Aguilera et al., 2011)

3.1.2. In case that the model is intended for decision analysis, what analytics the paper includes?

Expected utilities (EU) / Decision optimization (DO) / Value of information (VOI) / Value of control (VOC) / Other (O) / No decision analysis included (NA)

3.1.3. Describe shortly what results and in which format the authors present in the article.

3.1.4. What type(s) of validation method(s) is(are) used (options from Aguilera et al. 2011)?

Train & Test* (TT) / Cross Validation** (CV) / Expert evaluation*** (E) / Comparison to previous models (PM) / Sensitivity analysis (SA)**** / Goodness of fit***** (GF) / No validation (NV)

* The performance of a data-based model is evaluated against (an)other data set(s), not included in the modelling process at all.

** An x-fold repeated procedure, where x pieces of the data are left outside the modelling process one at a time and the performances of the resulting models are tested against the excluded data.

*** Expert judgement is used to evaluate how well the model performs in representing the functioning of the system (e.g. checking the output is reasonable and plausible in terms of the input).

**** Incl. e.g. entropy-based sensitivities (i.e. mutual information, i.e. “strengths of the links”) and value of information testing.

***** The model’s capacity to predict the same data that is used to learn or otherwise quantify the model.

3.2.2. If included in the analysis, describe shortly, how the authors validate, i.e. evaluate the performance and logic (rationality) of their model?

3.3. End use

3.3.1. Who are the intended end-users of the actual model?

The model developers themselves (MD) / Other scientists (OS) / Decision-makers (incl. planners and managers) (DM) / Stakeholders (SH) / Teachers (T) / Common public (CP) / Not clear (NC)

3.3.2. In case that the answer to 3.3.1 is something else than MD, is the use of the model intended to be facilitated by the modellers?

Yes (Y) / No (N) / Not applicable (NA)

3.3.3. Describe shortly the intended end use of the model:

3.3.4. If the model is intended to be used by the non-modellers (with or without facilitator), has this been tested?

Yes (Y) / No (N) / Not applicable (NA)

3.3.5. Who are the intended end-users of the results produced by the model?

The model developers themselves (MD) / Other scientists (OS) / Decision-makers (incl. planners and managers) (DM) / Stakeholders (SH) / Teachers (T) / Common public (CP) / Not clear (NC)

3.3.7. Describe shortly the intended end use of the results provided by the model as described in the article:

**Discussion**

4.1. Pros and cons

4.1.1. What pros of the BN and the presented approach are mentioned?

4.1.2. What cons of the BN and the presented approach are mentioned?

4.2. Future development ideas

4.2.1. What future steps are suggested when it comes to the use of BNs for the corresponding work (especially in the ERA context)?
