# Supplementary material for: Bayesian Networks in Environmental Risk Assessment: A Review
Source: Integr Environ Assess Manag. 2020 Oct 6;17(1):62–78. doi: 10.1002/ieam.4332 (PMC7821106; doi:10.1002/ieam.4332)
Supplement: Supplementary file 5 — Supporting information. [file IEAM-17-62-s005.docx]

**Table S5.** Studies removed at the final step of the content analysis and rationale for exclusion.

| **Title** | **Authors** | **Year** | **Reason for exclusion** |
| --- | --- | --- | --- |
| Modelling seasonal habitat suitability for Wide-Ranging Species: Invasive wild pigs in northern Australia | Froese, J.G.; Smith, C.S.; Durr, P.A.; McAlpine, C.A.; Klinken, R.D.V. | 2017 | Not framed as ERA |
| Probabilistic Evaluation of Ecological and Economic Objectives of River Basin Management Reveals a Potential Flaw in the Goal Setting of the EU Water Framework Directive | Hjerppe, T.; Taskinen, A.; Kotamäki, N.; Malve, O.; Kettunen, J. | 2017 | Not framed as ERA |
| EBI: An index for delivery of ecosystem service bundles | Van Der Biest, K.; D'Hondt, R.; Jacobs, S.; Landuyt, D.; Staes, J.; Goethals, P.; Meire, P. | 2014 | Not framed as ERA / no environmental risk addressed |
| Assessing coastal sustainability: A Bayesian approach for modeling and estimating a global Index for measuring risk | Vitabile, S.; Farruggia, A.; Pernice, G.; Gaglio, S. | 2013 | Not framed as ERA / no environmental risk addressed |
| Collaborative decision-analytic framework to maximize resilience of tidal marshes to climate change | Thorne, K.M.; Mattsson, B.J.; Takekawa, J.; Cummings, J.; Crouse, D.; Block, G.; Bloom, V.; Gerhart, M.; Goldbeck, S.; Huning, B.; Sloop, C.; Stewart, M.; Taylor, K.; Valoppi, L. | 2015 | Not framed as ERA / no environmental risk addressed |
| Influence of urbanisation characteristics on the variability of particle-bound heavy metals build-up: A comparative study between China and Australia | Wijesiri, B.; Liu, A.; Gunawardana, C.; Hong, N.; Zhu, P.; Guan, Y.; Goonetilleke, A. | 2018 | Not framed as ERA |
| Climate change, cyanobacteria blooms and ecological status of lakes: A Bayesian network approach | Moe, S.J.; Haande, S.; Couture, R.-M. | 2016 | Not framed as ERA |
| A bayesian belief network approach to predict damages caused by disturbance agents | Radl, A.; Lexer, M.J.; Vacik, H. | 2017 | Not framed as ERA |
| A Bayesian belief network approach for assessing uncertainty in conceptual site models at contaminated sites | Thomsen, N.I.; Binning, P.J.; McKnight, U.S.; Tuxen, N.; Bjerg, P.L.; Troldborg, M. | 2016 | Not framed as ERA / no environmental risk addressed |
| Site-specific updating and aggregation of bayesian belief network models for multiple experts | Stiber, N.A.; Small, M.J.; Pantazidou, M. | 2004 | Not framed as ERA / no environmental risk addressed |
| Secondary extinctions in food webs: A Bayesian network approach | Eklöf, A.; Tang, S.; Allesina, S. | 2013 | No case study |
| Integration of interpretive structural modelling with Bayesian network for biodiesel performance analysis | Sajid, Z.; Khan, F.; Zhang, Y. | 2017 | Not framed as ERA / no environmental risk addressed |
| Groundwater quality assessment using data clustering based on hybrid Bayesian networks | Aguilera, P.A.; Fernández, A.; Ropero, R.F.; Molina, L. | 2013 | Not framed as ERA / no environmental risk addressed |
| Environmental Bioindication Studies by Bayesian Network with Use of Grey Heron as Model Species | Sujak, A.; Kusz, A.; Rymarz, M.; Kitowski, I. | 2017 | Not framed as ERA / no environmental risk addressed |
| Impact assessment of emission management strategies of the pharmaceuticals Metformin and Metoprolol to the aquatic environment using Bayesian networks | Brandmayr, C.; Kerber, H.; Winker, M.; Schramm, E. | 2015 | Not framed as ERA / no environmental risk addressed |
| Application of Bayesian network to the probabilistic risk assessment of nuclear waste disposal | Lee, C.-J.; Lee, K.J. | 2006 | Not framed as ERA/ no clear case study |
| Integration of wellbore pressure measurement and groundwater quality monitoring to enhance detectability of brine and CO2 leakage | Yang, Y.-M.; Dilmore, R.M.; Mansoor, K.; Buscheck, T.A.; Bromhal, G.S. | 2019 | Not framed as ERA / no environmental risk addressed |
| Local versus Regional Soil Screening Levels to Identify Potentially Polluted Areas | Boente, C.; Gerassis, S.; Albuquerque, M.T.D.; Taboada, J.; Gallego, J.R. | 2019 | Not framed as ERA |
| Consequence-based framework for buried infrastructure systems: A Bayesian belief network model | Kabir, G.; Balek, N.B.C.; Tesfamariam, S. | 2018 | Not framed as ERA / no environmental risk addressed |
| Accident risk-based life cycle assessment methodology for green and safe fuel selection | Khakzad, S.; Khan, F.; Abbassi, R.; Khakzad, N. | 2017 | Not framed as ERA / no environmental risk addressed |
| Robust vulnerability analysis of nuclear facilities subject to external hazards | Tolo, S.; Patelli, E.; Beer, M. | 2017 | Not framed as ERA / no environmental risk addressed |
| Using robust Bayesian network to estimate the residuals of fluoroquinolone antibiotic in soil | Li, X.; Xie, Y.; Li, L.; Yang, X.; Wang, N.; Wang, J. | 2015 | Not framed as ERA / no environmental risk addressed |
| A general risk-based adaptive management scheme incorporating the Bayesian Network Relative Risk Model with the South River, Virginia, as case study | Landis, W.G.; Markiewicz, A.J.; Ayre, K.K.; Johns, A.F.; Harris, M.J.; Stinson, J.M.; Summers, H.M. | 2017 | No case study |
| A probabilistic model for accidental cargo oil outflow from product tankers in a ship-ship collision | Goerlandt, F.; Montewka, J. | 2014 | Not framed as ERA / no environmental risk addressed |
| Assessing model structure uncertainty through an analysis of system feedback and Bayesian networks | Hosack, G.R.; Hayes, K.R.; Dambacher, J.M. | 2008 | Not framed as ERA |
| A human-environmental network model for assessing coastal mitigation decisions informed by imperfect climate studies | Small, M.J.; Xian, S. | 2018 | Not framed as ERA |
| Multi-criteria decision assessments using Subjective Logic: Methodology and the case of urban water strategies | Moglia, M.; Sharma, A.K.; Maheepala, S. | 2012 | Not framed as ERA |
