# Supplementary material for: Bayesian Networks in Environmental Risk Assessment: A Review
Source: Integr Environ Assess Manag. 2020 Oct 6;17(1):62–78. doi: 10.1002/ieam.4332 (PMC7821106; doi:10.1002/ieam.4332)
Supplement: Supplementary file 6 — Supporting information. [file IEAM-17-62-s006.docx]

**Table S6.** Studies removed during the full text screening and reason for exclusion.

| **Title** | **Authors** | **Reason** | **Additional information** |
| --- | --- | --- | --- |
| Probabilistic risk assessment and decision support tools for the evaluation of oil transport in the gulf of Finland, North-eastern Baltic sea | Lehikoinen, A.; Luoma, E.; Hänninen, M.; Storgård, J.; Kuikka, S. | Conferece paper |  |
| Evaluation of the traffic increase in the gulf of Finland during the years 2007-2015 and the effect of the increase on the environment and traffic Chain activities | Lehikoinen, A.; Luoma, E.; Hanninen, M.; Storgard, J.; Kuikka, S. | Conferece paper |  |
| Uncertainty about uncertainty within a stakeholder group | Chan, T.; McShane, P.; Ross, H. | Conferece paper |  |
| Bayesian inference for predicting potential oil spill related ecological risk | Aps, R.; Fetissov, M.; Herkül, K.; Kotta, J.; Leiger, R.; Mander, Ü.; Suursaar, Ü. | Conferece paper |  |
| A risk-based approach to the improved understanding and management of denitrification in urban stormwater treatment wetlands | Overall, R.A.; Grace, M.R.; Pollino, C.A.; Hart, B.T. | Conferece paper |  |
| Development of an interdisciplinary bayesian network tool for catchment management in the Solomon Islands | Chan, T.U.; Ross, H.; Powell, B.; Hoverman, S. | Conferece paper |  |
| Development of criteria for simplifying ecological risk models | Thomas, C.; Hart, B.; Nicholson, A.; Grace, M.; Brodie, J.; Pollino, C. | Conferece paper |  |
| Development and application of a bayesian decision support tool to assist in the management of an endangered species | Pollino, C.A.; White, A.K. | Conferece paper |  |
| Regional and Temporal Transferability of Multivariable Flood Damage Models | Wagenaar, D.; Lüdtke, S.; Schröter, K.; Bouwer, L.M.; Kreibich, H. | "Environment" does not refer to the living environment (both humans and wildlife) |  |
| Sequential information gathering schemes for spatial risk and decision analysis applications | Eidsvik, J.; Martinelli, G.; Bhattacharjya, D. | "Environment" does not refer to the living environment (both humans and wildlife) & no ERA reference |  |
| A Bayesian network approach to study hydromorphological modifications over space and time in the framework of a sustainable river restoration project: the “Lac des Gaves” case study | Yassine, R.; Pérès, F.; Roux, H.; Cassan, L.; Frysou, O. | Conference proceedings, no model presented (paper presents next steps of the project) |  |
| Decision support tool employing Bayesian risk framework for environmentally safe shipping | Gyftakis, S.; Koromila, I.; Giannakopoulos, T.; Nivolianitou, Z.; Charou, E.; Perantonis, S. | Book chapter |  |
| Incorporation of human factors into ship collision risk models focusing on human centred design aspects | Sotiralis, P.; Ventikos, N.P.; Hamann, R.; Golyshev, P.; Teixeira, A.P. | Book chapter | Only collision risk |
| Bayesian network to predict environmental risk of a possible ship accident | Nivolianitou, Z.S.; Koromila, I.A.; Giannakopoulos, T. | "Environment" does not refer to the living environment (both humans and wildlife) |  |
| Environmental risk assessment for the aegean sea | Koromila, I.; Nivolianitou, Z.; Perantonis, S.; Giannakopoulos, T.; Charou, E.; Gyftakis, S.; Spyrou, K. | Book chapter |  |
| Bayesian network to predict environmental risk of a possible ship accident | Nivolianitou, Z.S.; Koromila, I.A.; Giannakopoulos, T. | Conferece paper |  |
| Estimating hazardous concentrations by an informative Bayesian approach | Ciffroy, P.; Keller, M.; Pasanisi, A. | No BN |  |
| An application of the integrated risk analysis methodology for the cooling systems of energy power plants | Léger, A.; Duval, C.; David, F.; Cordier, H. | Conferece paper |  |
| Overview of Bayesian network approaches to model gene-environment interactions and cancer susceptibility | Sua, C.; Andrewb, A.; Karagasb, M.; Borsuka, M.E. | Conferece paper |  |
| Coupling Bayesian networks and geospatial software for environmental risk assessment | Jolma, A.; Lehikoinen, A.; Helle, I. | Conferece paper |  |
| Bayesian inference in oil spill response management | Aps, R.; Sawano, N.; Hamada, S.; Fetissov, M. | Conferece paper |  |
| Graph-based analysis of nasopharyngeal carcinoma with bayesian network learning methods | Aussem, A.; Rodrigues De Morais, S.; Corbex, M.; Favrel, J. | Book chapter |  |
| A probabilistic approach to exposure risk assessment | Mutshinda, C.M.; Antai, I.; O'Hara, R.B. | No BN |  |
| An innovating application of Bayesian Networks: Global environmental risk assessment of the cold source system of a nuclear power plant | Dutfoy, A.; Pierlot, S.; Deleuze, G. | Conferece paper |  |
| Nasopharyngeal carcinoma data analysis with a novel Bayesian network skeleton learning algorithm | Aussem, A.; De Morais, S.R.; Corbex, M. | Conferece paper |  |
| An innovating application of Bayesian Networks: Global environmental risk assessment of the cold source system of a nuclear power plant | Dutfoy, A.; Pierlot, S.; Deleuze, G. | Conferece paper |  |
| Growth of the Decision Tree: Advances in Bottom-Up Climate Change Risk Management | Ray, P.A.; Taner, M.Ü.; Schlef, K.E.; Wi, S.; Khan, H.F.; Freeman, S.S.G.; Brown, C.M. | BN not used for ERA |  |
| Improving sustainability through usability | Duffy, V.G. | Book chapter |  |
| Exploring the utility of risk management as an integrating framework for the development and application of water resource planning tools | Cuddy, S.M.; Pollino, C.A. | Conferece paper |  |
| On the quantification of intertest variability in ecotoxicity data with application to species sensitivity distributions | Hickey, G.L.; Craig, P.S.; Luttik, R.; de Zwart, D. | No BN |  |
| Coupled Bayesian Networks and recursive partitioning method for wetland ecological modelling | Fu, B.; Pollino, C.A.; Merritt, W.; Capon, S. | Conferece paper |  |
| The development of an integrated systems model for balancing coral reef health, land management and tourism risks on the Great Barrier Reef | Thomas, C.R.; Gordon, I.J.; Wooldridge, S.; Van Grieken, M.; Marshall, P. | Conferece paper |  |
| River restoration using simple decision support tools in the Lower Snowy River | Glendining, N.S.; Pollino, C.A. | Conferece paper |  |
| How believable is your BBN? | Kuhnert, P.M.; Hayes, K.R. | Conferece paper |  |
| Making species salinity sensitivity distributions reflective of naturally occurring communities: Using rapid testing and Bayesian statistics | Hickey, G.L.; Kefford, B.J.; Dunlop, J.E.; Craig, P.S. | No BN |  |
| A consistent framework for knowledge integration to support integrated catchment management | Holzkämper, A.; Surridge, B.; Paetzold, A.; Kumar, V.; N. Lerner, D.; Maltby, L.; Wainwright, J.; W. Anderson, C.; Harris, R. | Conferece paper |  |
| Rats and rice: Belief network models of rodent control in the rice fields of Cambodia | Smith, C.; Russell, I.; King, C. | Conferece paper |  |
| Dynamic Bayesian networks based approach for risk analysis of subsea wellhead fatigue failure during service life | Chang, Y.; Wu, X.; Zhang, C.; Chen, G.; Liu, X.; Li, J.; Cai, B.; Xu, L. | "Environment" does not refer to the living environment (both humans and wildlife); BN not used for ERA | Risk of subsea wellhead failure only |
| An integrated approach for real-time hazard mitigation in complex industrial processes | Rebello, S.; Yu, H.; Ma, L. | "Environment" does not refer to the living environment (both humans and wildlife); BN not used for ERA | Industrial risks |
| A methodological framework for identifying potential sources of soil heavy metal pollution based on machine learning: A case study in the Yangtze Delta, China | Jia, X.; Hu, B.; Marchant, B.P.; Zhou, L.; Shi, Z.; Zhu, Y. | No BN | No BN (only naive bayes classifiers) |
| Application of the Public Health Exposome Framework to Estimate Phenotypes of Resilience in a Model Ohio African-American Women’s Cohort | Cifuentes, P.; Reichard, J.; Im, W.; Smith, S.; Colen, C.; Giurgescu, C.; Williams, K.P.; Gillespie, S.; Juarez, P.D.; Hood, D.B. | "Environment" does not refer to the living environment (both humans and wildlife); BN not used for ERA | No environmental risks, human health perspective |
| Development of a Bayesian network model for optimal site selection of electric vehicle charging station | Hosseini, S.; Sarder, M.D. | BN not used for ERA | Site selection for electric vehicles |
| Uncertanty Analysis of Project Emissions | Abdi, A.; Taahipour, S. | Conferece paper |  |
| Probabilistic Framework for Integrating Multiple Data Sources to Estimate Disaster and Failure Events and Increase Situational Awareness | Lee, C.; Tien, I. | No BN |  |
| Marine transportation risk assessment using Bayesian Network: Application to Arctic waters | Baksh, A.-A.; Abbassi, R.; Garaniya, V.; Khan, F. | BN not used for ERA | Accident risk only |
| Bayesian network-based risk analysis methodology: A case of atmospheric and vacuum distillation unit | Zhang, J.; Cai, B.; Mulenga, K.; Liu, Y.; Xie, M. | "Environment" does not refer to the living environment (both humans and wildlife) | system risk analysis, env risks not considered |
| A robust risk assessment methodology for safety analysis of marine structures under storm conditions | Abaei, M.M.; Arzaghi, E.; Abbassi, R.; Garaniya, V.; Chai, S.; Khan, F. | "Environment" does not refer to the living environment (both humans and wildlife) | Risk on built structures |
| Detection and attribution of nitrogen runoff trend in China's croplands | Hou, X.; Zhan, X.; Zhou, F.; Yan, X.; Gu, B.; Reis, S.; Wu, Y.; Liu, H.; Piao, S.; Tang, Y. | BN not used for ERA |  |
| A comparative analysis of bayesian network and ARIMA approaches to malaria outbreak prediction | Hasan, A.H.M.I.; Haddawy, P.; Lawpoolsri, S. | Book chapter |  |
| Is planting trees the solution to reducing flood risks? | Carrick, J.; Abdul Rahim, M.S.A.B.; Adjei, C.; Ashraa Kalee, H.H.H.; Banks, S.J.; Bolam, F.C.; Campos Luna, I.M.; Clark, B.; Cowton, J.; Domingos, I.F.N.; Golicha, D.D.; Gupta, G.; Grainger, M.; Hasanaliyeva, G.; Hodgson, D.J.; Lopez-Capel, E.; Magistrali, A.J.; Merrell, I.G.; Oikeh, I.; Othman, M.S.; Ranathunga Mudiyanselage, T.K.R.; Samuel, C.W.C.; Sufar, E.K.H.; Watson, P.A.; Zakaria, N.N.A.B.; Stewart, G. | Review paper |  |
| Knowledge engineering framework to quantify dependencies between epidemiological and biomolecular factors in breast cancer | Innokenteva, I.; Hammer, R.; Shin, D. | Conference paper |  |
| Introduction of Bayesian network in risk analysis of maritime accidents in Bangladesh | Rahman, S. | Conference paper |  |
| Personalization of Infectious Disease Risk Prediction: Towards Automatic Generation of a Bayesian Network | Vinarti, R.A.; Hederman, L. | Conference paper |  |
| Prioritizing safety critical human and organizational factors of EER systems of offshore installations in a harsh environment | Norazahar, N.; Khan, F.; Veitch, B.; MacKinnon, S. | "Environment" does not refer to the living environment (both humans and wildlife); BN not used for ERA | Operational risks |
| Risk Analysis on Leakage Failure of Natural Gas Pipelines by Fuzzy Bayesian Network with a Bow-Tie Model | Shan, X.; Liu, K.; Sun, P.-L. | "Environment" does not refer to the living environment (both humans and wildlife) | Natural gas leakage risk |
| Socioeconomic and air pollution correlates of adult asthma, heart attack, and stroke risks in the United States, 2010–2013 | Cox, L.A.T. | "Environment" does not refer to the living environment (both humans and wildlife) | Air pollution, human health perspective |
| Fault tree analysis of oil and gas distillation tower and application of Bayesian Networks | Nassaj, A.; Barabady, J. | Conferece paper |  |
| Development of Generic Tools for Coastal Early Warning and Decision Support | Bogaard, T.; De Kleermaeker, S.; Jaeger, W.S.; Van Dongeren, A. | Conferece paper |  |
| Leak Prediction Model for Water Distribution Networks Created Using a Bayesian Network Learning Approach | Leu, S.-S.; Bui, Q.-N. | "Environment" does not refer to the living environment (both humans and wildlife) | Pipe leakage risk only |
| Bayesian networks in infectious disease ecoepidemiology | Lau, C.L.; Smith, C.S. | Review paper |  |
| Application of Molecular Typing Results in Source Attribution Models: The Case of Multiple Locus Variable Number Tandem Repeat Analysis (MLVA) of Salmonella Isolates Obtained from Integrated Surveillance in Denmark | de Knegt, L.V.; Pires, S.M.; Löfström, C.; Sørensen, G.; Pedersen, K.; Torpdahl, M.; Nielsen, E.M.; Hald, T. | No BN |  |
| Risk analysis of emergent water pollution accidents based on a Bayesian Network | Tang, C.; Yi, Y.; Yang, Z.; Sun, J. | "Environment" does not refer to the living environment (both humans and wildlife) | Risk of accidents |
| Public discourse on environmental pollution &amp; health in Korea: Tweets following the Fukushima nuclear accident | Kim, S.-H.; Ha, Y.-I.; Cha, M.; Lee, J.; Kim, B.-J.; Lee, D.-M. | Conference paper |  |
| A comprehensive model for oil spill liability estimation | Boehm, P.; Morrison, A.M.; Semenova, S.; Kashuba, R.; Ahnell, A.; Monti, C. | Conference paper |  |
| Probabilistic vulnerability assessment of chemical clusters subjected to external Acts of Interference | Argenti, F.; Landucci, G.; Reniers, G. | BN not used for ERA | Risk due to deliberate attacks |
| Beyond QMRA: Modelling microbial health risk as a complex system using Bayesian networks | Beaudequin, D.; Harden, F.; Roiko, A.; Stratton, H.; Lemckert, C.; Mengersen, K. | Review paper |  |
| Risk assessment of wind turbines: Transition from pure mechanistic paradigm to modern complexity paradigm | Ashrafi, M.; Davoudpour, H.; Khodakarami, V. | "Environment" does not refer to the living environment (both humans and wildlife) | Operational risks |
| Reducing the risk of house loss due to wildfires | Penman, T.D.; Nicholson, A.E.; Bradstock, R.A.; Collins, L.; Penman, S.H.; Price, O.F. | "Environment" does not refer to the living environment (both humans and wildlife) | Risks to property due to wildfires |
| Risk assessment of Arctic drilling waste management operations based on Bayesian Networks | Ayele, Y.Z.; Barabady, J.; López Droguett, E. | Conference paper |  |
| Influence of fuels, weather and the built environment on the exposure of property to wildfire | Penman, T.D.; Collins, L.; Syphard, A.D.; Keeley, J.E.; Bradstock, R.A. | "Environment" does not refer to the living environment (both humans and wildlife) | Risks to property from wildfires |
| Improvement and application of modeling method for food microbial risk assessment | Liu, L.; Gao, Y.; Wang, D. | Fulltext not accessible | Written in Chinese |
| Using Bayesian networks to explore the role of weather as a potential determinant of disease in pigs | McCormick, B.J.J.; Sanchez-Vazquez, M.J.; Lewis, F.I. | BN not used for ERA |  |
| Assessment of debris flow hazards using a Bayesian Network | Liang, W.-J.; Zhuang, D.-F.; Jiang, D.; Pan, J.-J.; Ren, H.-Y. | "Environment" does not refer to the living environment (both humans and wildlife) | Debris flow hazard risk |
| Use of Bayesian networks to dissect the complexity of genetic disease: Application to the Genetic Analysis Workshop 17 simulated data | Kang, J.; Zheng, W.; Li, L.; Lee, J.S.; Yan, X.; Zhao, H. | BN not used for ERA |  |
| Icemaster - A toolbox for planning of arctic offshore operations | Hüffmeier, J.; Sandkvist, J.; Forsman, B.; Rafstedt, J. | Conference paper |  |
| Integrated landscape management methodology: An application of Bayesian Belief Networks (BBNs) to the UNESCO landscape of the Diamantina (Ferrara, Italy) | De Grassi, M.; Naticchia, B.; Gissi, E. | Conference proceedings |  |
| Using bayesian belief networks to predict radioactive releases after a severe accident in a BWR | Knochenhauer, M.; Frid, W.; Johansson, M. | Conference paper |  |
| Bayesian networks for decision analyses - An application to irrigation system selection | Robertson, D.; Wang, Q.J. | Fulltext not available |  |
| Applying Probabilistic Reasoning to Environmental Risk Analysis | Liu, Kevin Fong-Rey; Lu, Che-Fan | Conference paper |  |
| Risk Assessment for Ecological Planning of Arid Inland River Basins Under Hydrological and Management Uncertainties | Gu, Jinjin; Li, Mo; Guo, Ping; Huang, Guohe | No BN |  |
| Coastal and estuarine ecological risk assessment: the need for a more formal approach to stressor identification | Newman, Michael C.; Zhao, Yuan; Carriger, John F. | No BN | No model presented, review of options |
| Analysis of nasopharyngeal carcinoma risk factors with Bayesian networks | Aussem, A.; de Morais, S.R.; Corbex, M. | "Environment" does not refer to the living environment (both humans and wildlife) | Human health risk |
| Participatory modelling for stakeholder involvement in the development of flood risk management intervention options | Maskrey, Shaun A.; Mount, Nick J.; Thorne, Colin R.; Dryden, Ian | "Environment" does not refer to the living environment (both humans and wildlife) | Flood risks in general, environmental aspects not specified |
| Predicting arboviral disease emergence using Bayesian networks: A case study of dengue virus in Western Australia | Ho, S.H.; Speldewinde, P.; Cook, A. | "Environment" does not refer to the living environment (both humans and wildlife) | Human health risk |
| A Bayesian network model to assess the public health risk associated with wet weather sewer overflows discharging into waterways | Goulding, R.; Jayasuriya, N.; Horan, E. | "Environment" does not refer to the living environment (both humans and wildlife) | Human health risk |
| Bayesian network-based framework for exposure-response study design and interpretation | Orak, N.H.; Small, M.J.; Druzdzel, M.J. | "Environment" does not refer to the living environment (both humans and wildlife) | Human health risk |
| Bayesian network of risk assessment for a super-large dam exposed to multiple natural risk sources | Chen, Y.; Lin, P. | BN not used for ERA | Only risks to dam and not the environment |
| Assessing offshore wind turbine reliability and availability | Lazakis, I.; Kougioumtzoglou, M.A. | BN not used for ERA | Only system reliability |
| Predictive risk mapping of an environmentally-driven infectious disease using spatial Bayesian networks: A case study of leptospirosis in Fiji | Mayfield, H.J.; Smith, C.S.; Lowry, J.H.; Watson, C.H.; Baker, M.G.; Kama, M.; Nilles, E.J.; Lau, C.L. | "Environment" does not refer to the living environment (both humans and wildlife) | Human health risk |
| Modelling local areas of exposure to Schistosoma japonicum in a limited survey data environment | Araujo Navas, A.L.; Soares Magalhães, R.J.; Osei, F.; Fornillos, R.J.C.; Leonardo, L.R.; Stein, A. | "Environment" does not refer to the living environment (both humans and wildlife) | Human health risk |
| Using a Bayesian belief network model for early warning of death and severe risk of HFMD in Hunan province, China | Liao, Y.; Xu, B.; Liu, X.; Wang, J.; Hu, S.; Huang, W.; Luo, K.; Gao, L. | "Environment" does not refer to the living environment (both humans and wildlife) | Human health risk |
| Use of surrogate indicators for the evaluation of potential health risks due to poor urban water quality: A Bayesian Network approach | Wijesiri, B.; Deilami, K.; McGree, J.; Goonetilleke, A. | "Environment" does not refer to the living environment (both humans and wildlife) | Human health risk |
| A Bayesian network model for assessments of coastal inundation pathways and probabilities | Narayan, S.; Simmonds, D.; Nicholls, R.J.; Clarke, D. | "Environment" does not refer to the living environment (both humans and wildlife) | Flood risk |
| Applying nonparametric methods to analyses of short-term fine particulate matter exposure and hospital admissions for cardiovascular diseases among older adults | Tony Cox, L.A.; Liu, X.; Shi, L.; Zu, K.; Goodman, J. | "Environment" does not refer to the living environment (both humans and wildlife) | Human health risk |
| Climate Change Adaptation to Heat Risk at the Local Level: A Bayesian Network Analysis of Local Land-Use Plan Implementation | Donner, J.; Sprondel, N.F.; Köppel, J. | "Environment" does not refer to the living environment (both humans and wildlife) | Heat risk, human health perspective |
| Part II: Quantitative Evaluation of Choices Used in Setting Noncancer Chronic Human Health Reference Values Across Organizations | Holman, E.; Francis, R.; Gray, G. | "Environment" does not refer to the living environment (both humans and wildlife) | Human health risk |
| Assessing the Resilience of Coastal Systems: A Probabilistic Approach | Schultz, M.T.; Smith, E.R. | "Environment" does not refer to the living environment (both humans and wildlife) | Risks on the built environment |
| Utility of Bayesian networks in QMRA-based evaluation of risk reduction options for recycled water | Beaudequin, D.; Harden, F.; Roiko, A.; Mengersen, K. | "Environment" does not refer to the living environment (both humans and wildlife) | Human health risk |
| Modeling drinking water quality violations with Bayesian networks | Pike, W.A. | "Environment" does not refer to the living environment (both humans and wildlife) | Risk of system malfunctioning, human health perspective |
